# Circular visualisation of historical migration in England in the long eighteenth-century

**DOI:** 10.1016/j.heliyon.2020.e05490

**Published:** 2020-11-19

**Authors:** Stuart Gietel-Basten

**Affiliations:** Division of Social Science, The Hong Kong University of Science and Technology, Clear Water Bay, Kowloon, Hong Kong SAR, People's Republic of China

**Keywords:** History, Migration, England, Lincolnshire, Marriage, Parishes, Eighteenth-century, Nineteenth-century, Human geography, Human migration, Social geography, Sociology, Geography

## Abstract

Migration is a central component of both individual life-courses and macro-level demographic systems. In the absence of population registers and other surveillance systems, however, it is often difficult to measure. This is especially the case in historical populations. Compared to measures of fertility, nuptiality and mortality, then, migration processes are rather less studied. Recent studies in the English historical context have challenged long standing theoretical constructs concerning the relationship between migration and modernisation; gender and distance travelled; motivation for movement and the very nature of the movers themselves. Using a set of marriage registers for a large, agricultural county, this study explores intra-county migration among a predominantly young population over the period 1700 to 1836. The proportion of migrants is explored as well as the distance between ‘home’ and ‘marriage’ parishes. For perhaps the first time, chord diagrams are deployed for historical English migration data to visualise inter- and intra- regional/district migration. Although there are numerous limitations concerning the scope of the sample and the study, the evidence presented here broadly accords with recent studies of migration in pre-industrial England; and shows the potential to both use circular visualisation and exploit large scale samples of marriage registers to gain a further insight into a particular type of historical migration.

## 1 Introduction

### 1.1 Migration in pre-Victorian England

There is no doubt that migration has played a critical role in the development of overall population trends in pre-industrial and industrialising societies [1] such as England [2], as well as heavily influencing the elasticity of labour supply [3], the development of new ideas and attitudes [4] and the development of issues of social order - and opportunity under the auspices of the developing Poor Law system [5, 6]. As ‘urban sinks’ of lower birth rates and higher death rates, towns and cities largely relied on immigration to sustain their growth [7]. Migration to London [4, 8] and the industrial towns of the midlands and the north of England is perhaps the most well studied [9, 10]. The stereotype of the ‘emptying’ of rural parishes into such urban, industrialising sinks is a pervasive one. However, various studies have demonstrated that this is just one aspect of the migration experience in pre- and industrial England [11]. Pooley and Turnbull emphasise the primacy of local or regional migration, while others have argued for migration to be considered a fundamental component in linking towns to their hinterlands and the development of regional *pays* [2, 12]. This, in turn, was linked to ‘betterment migration’, where servants, apprentices and young landholders move as part of actualising their aspirations to social and economic improvement [13]. On the other hand, the work of Pooley and Clark, utilising thousands of individual family genealogies, suggests that migration during the eighteenth- and nineteenth-centuries was as much concerned with young adults moving together in family groups as aspiring individuals [11].

Linking these experiences to broader theoretical constructions of migration, it can be suggested - at least from the influential work of Pooley and Turnbull - that there is relatively little support for Zelinsky's notion of modernisation going hand-in-hand with an expanding geographical extent of mobility [14]. Similarly, the conventional theoretical frameworks set out by Ravenstein [15] in the later-nineteenth-century often do not match up to the recorded historical experience. The work by Pooley and Turnbull [11], for example, contradicted the views that males moved over longer distances than females and rural dwellers were far more likely to move than their urban counterparts. Rather, as Schwartz observes [16] ‘these life histories show that women and men were more like fellow travelers than the notion of separate spheres would suggest, resembling each other in the distances they moved and in age, marital status, and family position’.

The life histories studied by Pooley and Turnbull also reveal something of the motivation behind migration. Again, the stereotypical view is that work was the predominant factor - either as a necessity or seeking betterment and the realisation of aspiration. However, the life histories suggest that work alone was the sole motivation in only a minority of the cases studied. Rather, ‘the decision to move was influenced not merely by employment but also by considerations of marriage and housing, as well as one's family position, age, occupation, and other factors’.

Expanding on one such factor, marriage is widely held to be an important feature in shaping the migration profile of both historical and contemporary populations in England [17] and elsewhere [13, 18, 19, 20]. The contexts, distances and motivations for such movements are, of course, as varied as the populations under study. In the pre-industrial English context, though, the relatively few studies of the topic suggest that marriages occurred primarily between couples from the same parish. For example, in a group of Oxfordshire villages in the second half of the 18th century, 65 percent of marriages involved partners who lived in the same parish [21]. For marriages occurring where bride and groom hailed from different parishes, it has been suggested that these ‘marriage moves’ were ‘mostly over quite short distances’ [22]. However, there lies something of a tension between the notion of a relatively immobile population within a comparatively stable rural economy and a ‘face-to-face’ society [23] compared to a more dynamic, moving population - albeit travelling over small distances - as displayed in the genealogies of Pooley and Turnbull as well as other studies [24]. It is in this context that a comparative context is extremely valuable. Comparing different parts of the country not only allow for a broader empirical corpus of evidence of migration flows, but also allow the researcher to explore other potential systems and constructs which may be playing a role in shaping possible differentials. For example, Malcolmson [25] observes that in communities where geographical and social mobility is limited, social customs may be ever more deeply entrenched, potentially leading to a self-reinforcing mechanism.

### 1.2 Lincolnshire and it's administrative units

Lincolnshire is a ceremonial county situated in the East Midlands of England. The 1831 census estimated that the population of Lincolnshire was 317,465 [26]. According to the ‘Penny Cyclopedia’ written in 1839, ‘Lincolnshire is almost entirely an agricultural county’ [27]. ‘Of the 79,535 males twenty years and upwards’, it continues, ‘only 167 are employed in manufacturing machinery, while 45,272 are engaged in agricultural pursuits, 32,167 of which number are labourers’. In 1831, the largest settlements in the county were the city of Lincoln (population 11,843); Grantham (10,780), Boston (11,240) and Stamford (5,837).

**Figure 1:**
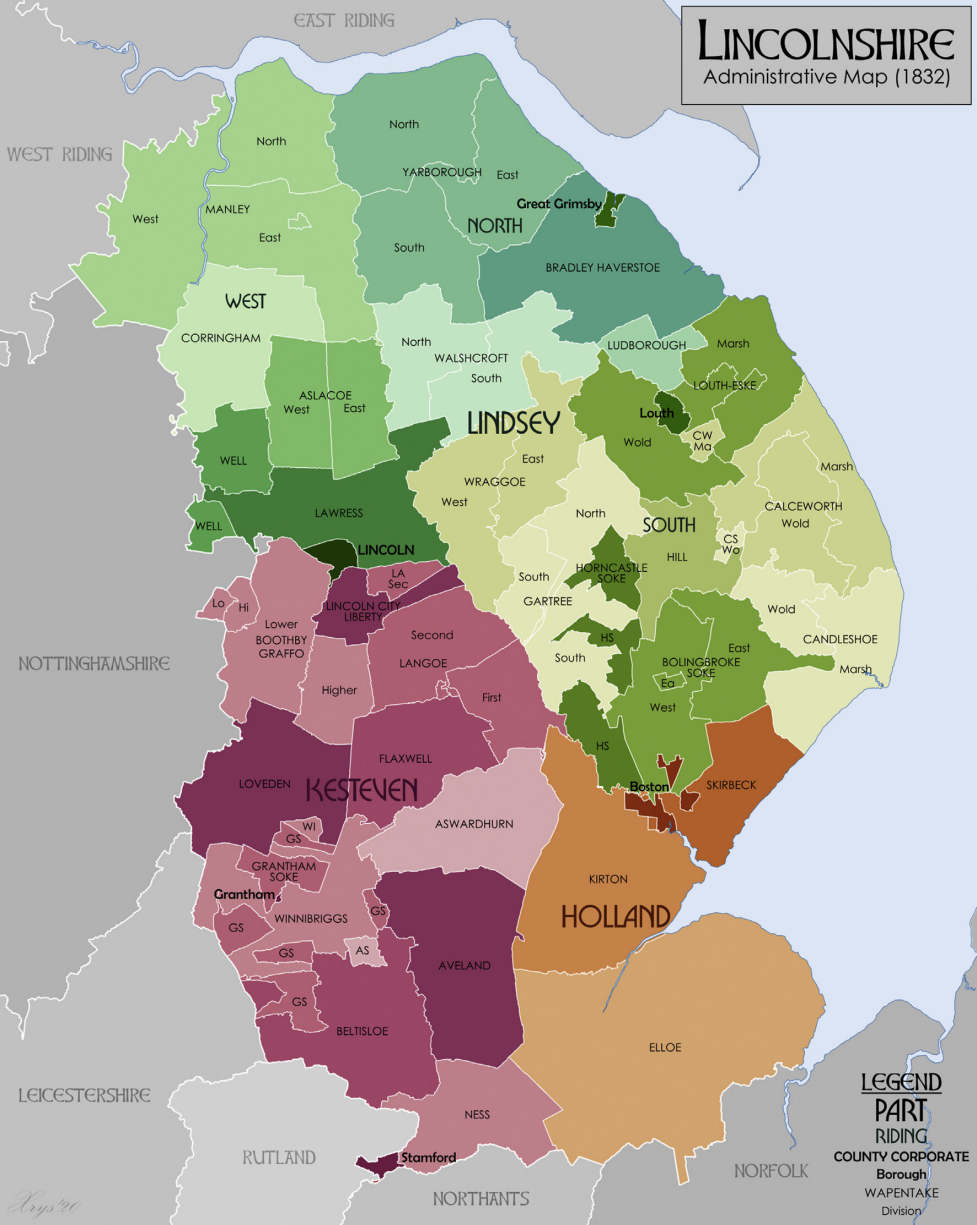
Administrative map of Lincolnshire in 1832 showing Wapentakes and Divisions. Also showing extant Boroughs and the County Corporate of Lincoln. Source: Reproduced from Wikimedia Commons under Creative Commons Attribution-Share Alike 4.0 International license [CC BY-SA 4.0]. Originally produced by WikiMedia user *XrysD.* Notes: Wapentake boundaries from University of Nottingham English Place Name Society. Source data for parish boundaries - Kain, R.J.P., and Oliver, R.R. (2001) "Historic parishes of England and Wales". Unit names and Borough Boundaries from Vision of Britain website and The First Report of the Commissioners Appointed to Inquire into the Municipal Boundaries in England and Wales, Part IV EASTERN CIRCUITS (1835).

The county was divided into three main ‘regions’, or ‘ridings’ (in a similar vein to Yorkshire) (see Figure 1). This term displays the Viking heritage of the county, as ‘riding’ is derived from the Norse *þriðjungr*, pertaining to a ‘third’ (especially of a county). These were known as Kesteven, Holland and Lindsey - the latter being informally divided into North, West and South Lindsey. Lincoln, Boston, Stamford and Grantham also operated as boroughs somewhat independently of the ridings [28]. For the purposes of analysis in this study, we refer to these ridings (and sub-divided ridings) as ‘regions’ within Lincolnshire. The next administration level in Lincolnshire was known as the ‘wapentake’. This unit of analysis, with its name rooted in Danish history, has been broadly suggested to correspond to the southern English ‘hundred’, although other studies have suggested the rather more unique particularities of the wapentake [29]. While these were very important administrative units in earlier centuries, their significance had largely waned in the post-medieval period. Despite this, they were still used for certain administrative reporting - for example in determining censal areas in the early nineteenth-century [30]. They also serve as a helpful intermediate level of analysis between county and parish.

Finally, the parish was, perhaps, the most significant administrative unit for the day-to-day lives of residents of Lincolnshire in the eighteenth- and early-nineteenth century. The parish - and the church - was the cornerstone of the established church and the *de facto* seat of local authority in most settings [31]. Parishes were responsible for various functions including the regulation of welfare and poverty through the poor law [5] as well as regulating settlement and migration [6]; levying taxation through church rates [32] as well as other responsibilities such as for policing and maintenance of roads [33].

### 1.3 Marriage registers

The registration of baptisms, weddings and funerals (as a proxy for the demographic events of births, marriages and deaths) was also an important function of the parish, especially before the onset of civil registration in 1837. The Anglican Church did not have a monopoly on the performance and registration of baptisms and funerals [34, 35] with non-conformist chapels able to perform such rites. Indeed, the number of such chapels grew significantly over the course of the eighteenth-century, especially in urbanising and industrializing parts of the country. This has led many to suggest that the study of demographic phenomena at the turn of the nineteenth-century is especially challenging [36, 37]. However, after the "Act for the Better Preventing of Clandestine Marriage" (popularly known as Lord Hardwicke's Marriage Act) (26 Geo. II. c. 33) came into force in 1754, all marriages were required to be performed in the local Anglican church [38, 39, 40]. Apart from the presence of lost registers, then, we may assume that the marriage record of the Anglican church in England - especially away from border areas - should represent a near complete record of marriages (unlike baptism and burial registers).

The majority of marriages were preceded by the reading of ‘banns’ - public announcements of the intended marriage for three consecutive weeks in home parishes of both the bride and groom during which any objections could be made. In marriage registers, it was common practice to note the ‘home parish’ of the bride and/or groom if it was different from the parish in which the ceremony occurred. This was often accompanied by reference to a ‘certificate’ which would demonstrate that the banns were read in the external parish and no objections were received.

As such, marriage registers represent an important means of determining migration in the pre-censal era. Indeed, they have been referred to as ‘one of the best sources for studying non-elite mobility’ [41]. Of course, such migration is only noted as a one-step process relatively early on in life. In the mid-eighteenth-century and early nineteenth-century, for example, the age of first marriage was around 25–26 for males and slightly less for females (as reported in various studies of the period) [42, 43]. In response to this, various studies have argued that marriage registers represent a significant under-count of total migration - especially when compared to, for example, baptism registers which may sometimes list the home parish of parents [24, 44]. While this last point about under-representation of total migration is intuitive and well taken, it should be specifically noted that the analysis of *total* migration can be distinct from a more narrowly focussed study on migration either before marriage, or for the purposes of marriage itself. This, then, is the focus of this study.

In this study, then, the primary research question here is to explore the characteristics of migration before and for marriage in Lincolnshire over the period 1700 to 1836. In particular, the research questions under consideration are (a) what percentage of the population married outside of their own parish, and what were the distances between ‘home’ and ‘marriages’ parishes. Based on the foregoing literature review, one may hypothesise that such migration accounted for the minority of overall marriages, and that the distance travelled was relatively modest. These data allow us to move on to perform some more exploratory, descriptive analysis of the patterns of migration in Lincolnshire over this period, and attempt to draw some preliminary conclusions and possible avenues for further study.

## 2 Materials and methods

**Figure 2:**
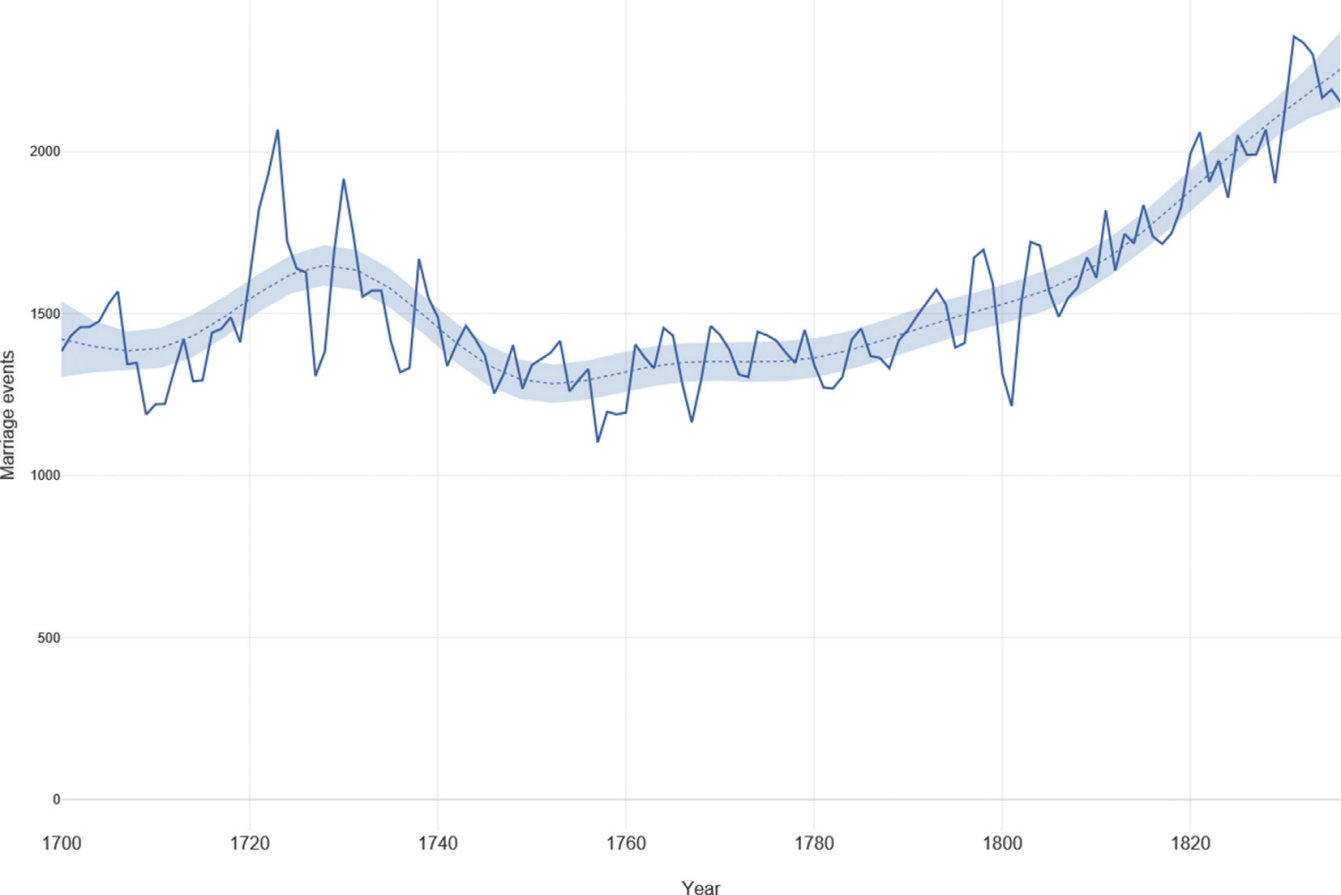
Number of marriage events per year in sample of 496 parishes (with confidence interval). Source: Lincolnshire marriage indexes.

Our sample covers data from 496 parishes (out of the total of 629) in the historical county of Lincolnshire for the period 1700 to 1837. A full list of the parishes and total events can be found in the Supplementary Information. In total, this represents 243,772 unique marriage events. Note that each marriage is counted as an individual event for each person; so to translate this figure to the number of *wedding ceremonies*, the figure is divided by two. Figure 2 shows the number of marriages per year for the total sample. The figure shows a pronounced increase over the late eighteenth- and into the early-nineteenth century. This, however, is likely to reflect the circumstances of overall population growth in the eighteenth-century, rather than any skewness in the data itself [45, 46, 47].

**Figure 3:**
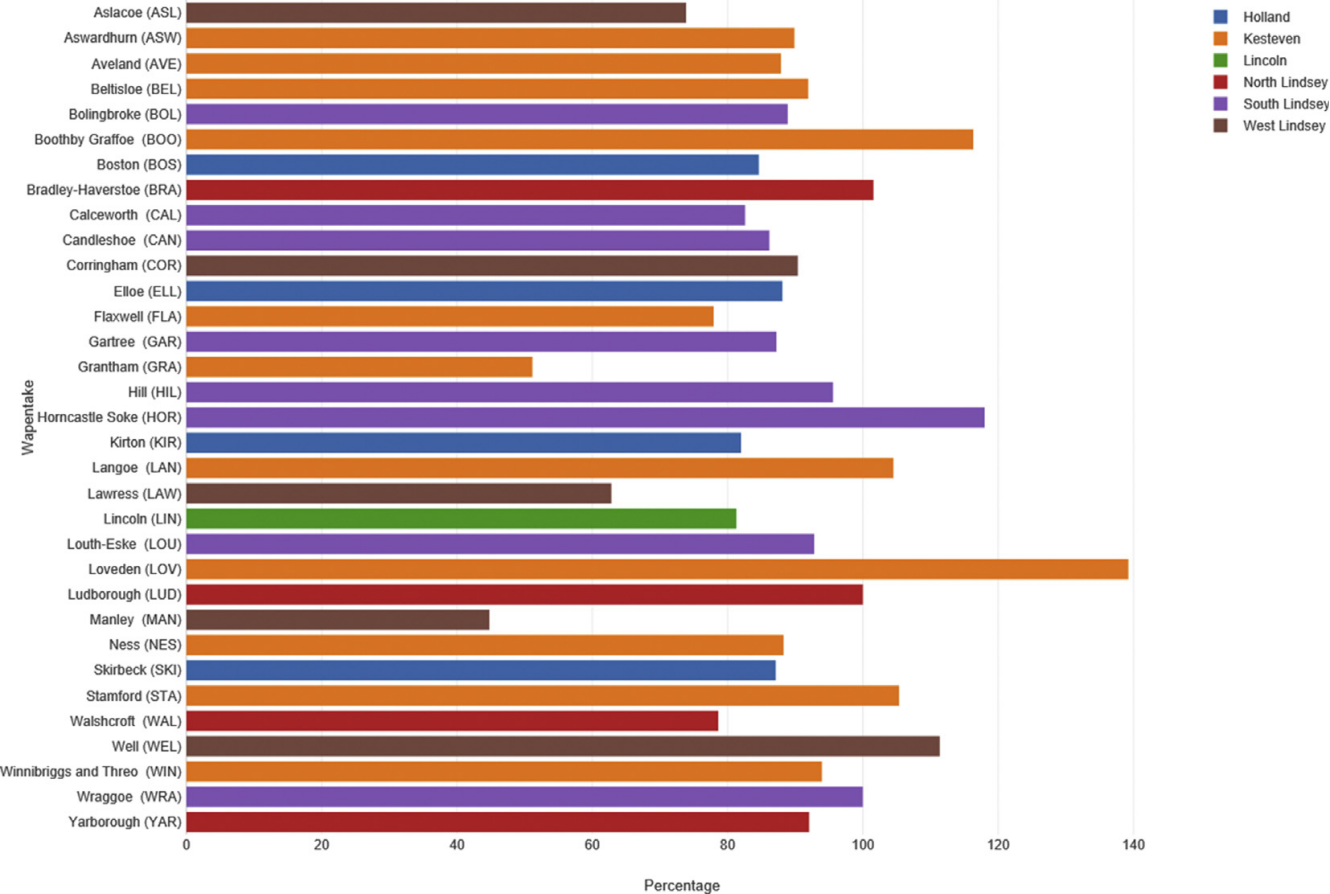
Coverage of marriages in sample of 496 parishes expressed as percentage of marriages in Parish Register Abstracts. Source: Lincolnshire marriage indexes [30, 48].

Given the heterogeneity in the population sizes of parishes, it is arguably more important to compare the number of events rather than the total number of parishes. This is only possible after 1801, when ‘Parish Register Abstracts’ [PRA] of baptisms, funerals and marriages by hundred/wapentake/borough were published alongside the Census. In order to make a comparison, three years were chosen at random (1811, 1821 and 1829), and the number of marriages in the sample summed and compared to the number of marriages in the PRAs [30, 48]. For the whole sample there were 5,740 marriages for the years combined, compared to 6,667 for the PRA count - equating to 86.0%. The number of marriages in the sample as a percentage by wapentake/borough/city of the PRA total is represented in Figure 3. Clearly, for some wapentakes such as Grantham/Manley, coverage of the sample is lower. Note that in some wapentakes the percentage is greater than 100. This is due to under-counting in the PRA. Indeed, we should also be aware of the data limitations of the PRA, which have been covered elsewhere [49]. Indeed, in a study which compared data from the PRA and register data from Shropshire, there were found to be some important deficiencies in the former including under-registration [50]. Taken together, though, we might suggest that the sample does not appear highly skewed by wapentake at a qualitative level. We return to the limitations of using a sample rather than full parish count in the Discussion section.

In order to avoid potential double counting, all instances of widowhood (as well as other duplicated entries) were removed. This left a total of 212,310 marriage events. In our analysis, we have used marriages *indices* which list only the date of marriage, names, parish and ‘comment.’ As shown below, the majority of cases had no comment. Under these circumstances we simply assume that both spouses hailed from the same parish (NB checking for systematic patterns of no comments were checked, for example to see if some parishes had no comments at all indicating a systematic inconsistency with transcription). This comment section referred to other pieces of information which would have been listed as relevant either in the register itself, or by the transcriber. In the case of the latter, this may be whether the marriage was transcribed from the original register or from a Bishop's Transcript, for example. If the bride or groom was widowed, or divorced, this would also be listed in the comments section (although the latter was not the case in any of the marriages in our sample). A typical transcribed comment reads as follows: “BT/Lic/SS Brumby/AD Sculthorpe/Incomplete date” (ID 337762, West Halton parish, 1706). This means that the entry was transcribed from a Bishop's Transcript; that the marriage occurred by license rather than by the more traditional multiple reading of banns [38]; one spouse with the initials SS hailed from Brumby, while the other spouse hailed from Sculthorpe. In order to process these comments it was necessary to first extract the initials of the first and last names of the spouses and match these to the initials in the comments section. Those which referred to a county apart from Lincolnshire were removed from the next stage of analysis (though will be discussed later on). The remaining places were then standardised to the list of parishes as derived from the UK Parish Locator software program [51] and, where appropriate, places within parishes were linked to their encompassing parishes. These parishes were then linked through to the list of wapentakes and regions derived from the historical maps of English jurisdictions produced by the Church of Jesus Christ of Latter Day Saints [52] and the PRAs [48]. The parishes in the marriage list were then updated with the corresponding wapentake and region (as well as the county where it was found to be outside of Lincolnshire).

Easting and northings for each parish were then derived from UK Parish Locator software program [51] and applied to the data set for both the parish where the marriage occurred (hereafter the ‘marriage parish’) as well as the parish listed in the comments section (hereafter the ‘home parish’). A simple Euclidean formula was then applied to calculate the distance between the two parishes. A more complex approach could have been taken using GIS, but given the short distances involved (i.e. for study within the county) this simple approach was deemed to suffice.

The analysis predominantly relies on descriptive statistics and figures. Box plots are produced to examine the distribution of distances between ‘home’ and ‘marriage’ parishes using R [53]. In order to show the patterns of migration, however, ‘chord plots’ are employed. These are circular plots which more easily enable the visualisation of multidimensional patterns of data [54]. In particular, they are especially helpful to show patterns of migration within a complex matrix of sending and receiving places. As such, they have been deployed in a number of demographic studies of migration [55, 56, 57, [71]. These diagrams are produced by creating a matrix of migrant flow from one unit to another (for example region to region, or district to district). The chord diagrams are then produced using the R package ‘*circlize*’ [59]. This study here is one of the very first to explore historical data using chord diagrams. The core domestic migration dataset, as well as the migration matrices and R code to replicate the chord diagrams can be found in the institutional depository.^1^

## 3 Results

### 3.1 Percentage of ‘marriage migrants’

**Figure 4:**
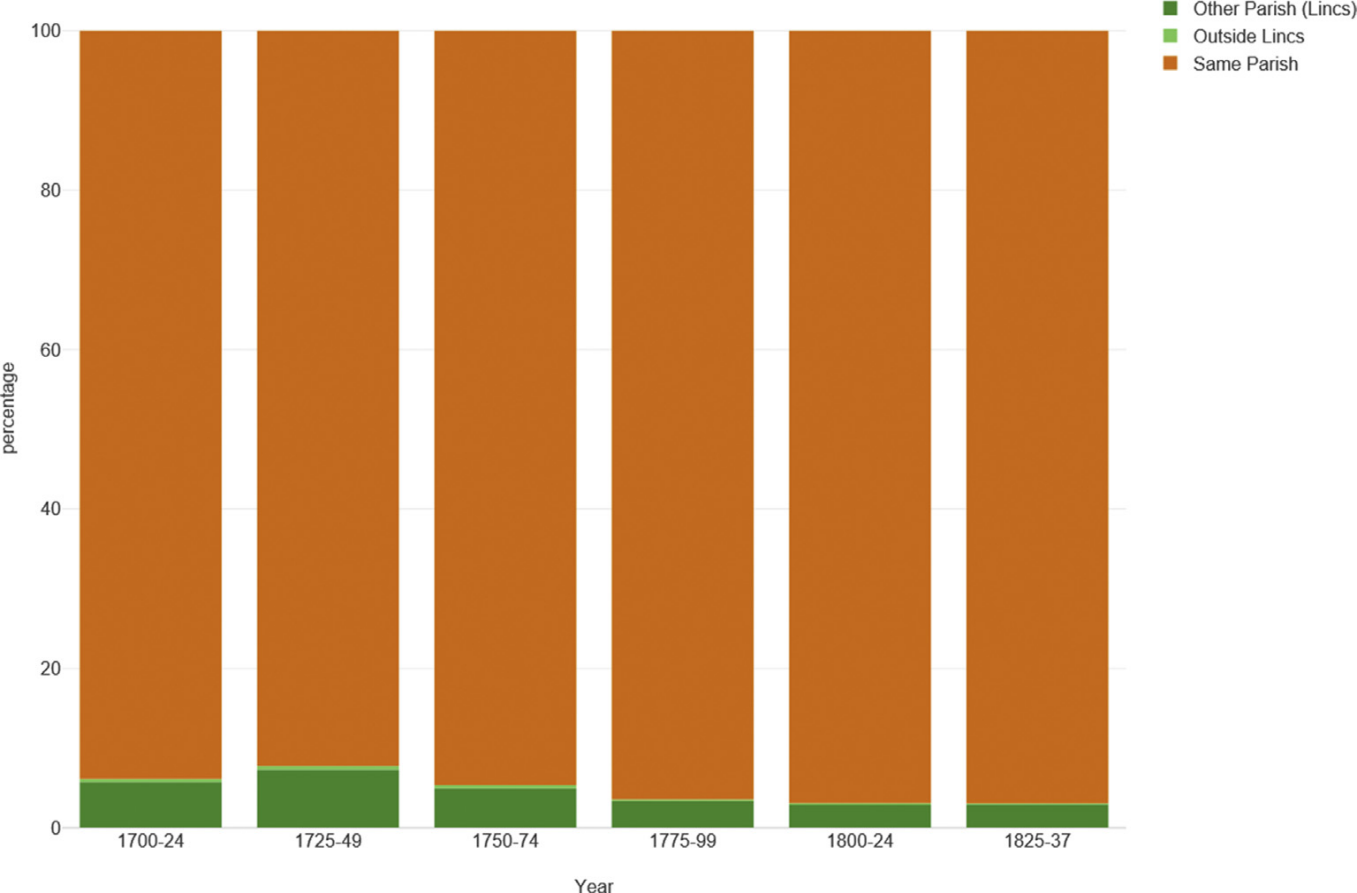
Distribution of marriages events by location of ‘home’ parish, sample of 484 Lincolnshire parishes, 1700–1836. Source: Lincolnshire marriage indexes.

Figure 4 shows the distribution of marriage events by the location of the ‘home’ parish for 25-year periods. Clearly, the vast majority of marriages - 94.9%, or between 92.2% (1725-49) and 96.9% (1800-24) over time - occur between couples who marry in their home parish. Of the remainder, 4.8% hailed from other parishes within Lincolnshire, while 0.5% came from beyond the county boundaries. We will return to this latter group in the discussion.

### 3.2 Distances between ‘home’ and ‘marriage’ parish

**Figure 5:**
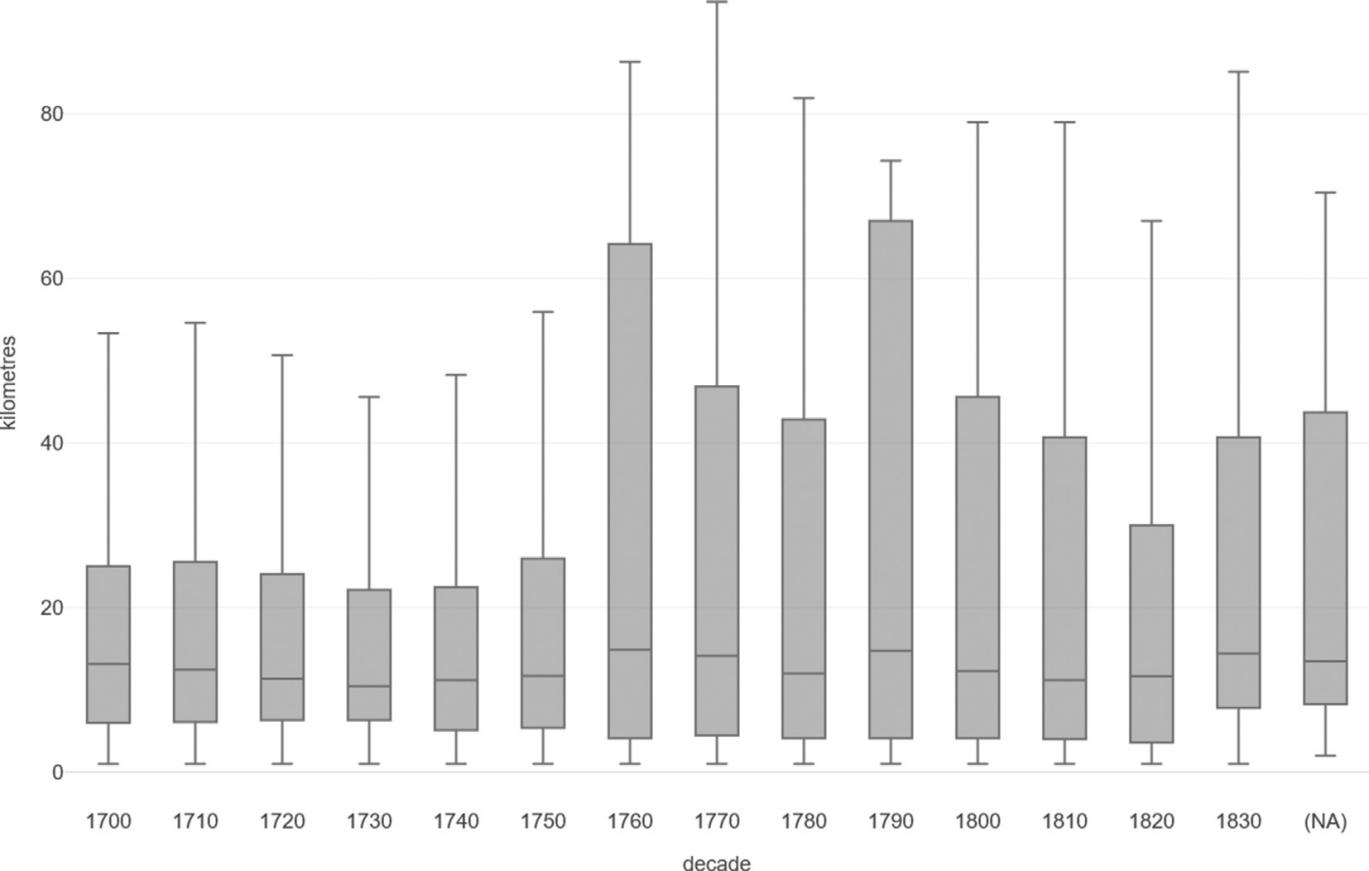
Boxplot of distance from ‘home parish’ to ‘marriage parish’, sample of 484 Lincolnshire parishes, 1700–1836. Source: Lincolnshire marriage indexes. Note: Based on N = 14,243 (where distance between co-ordinates is > 0). NB 1830 covers the period 1830-36. Outliers omitted.

**Figure 6:**
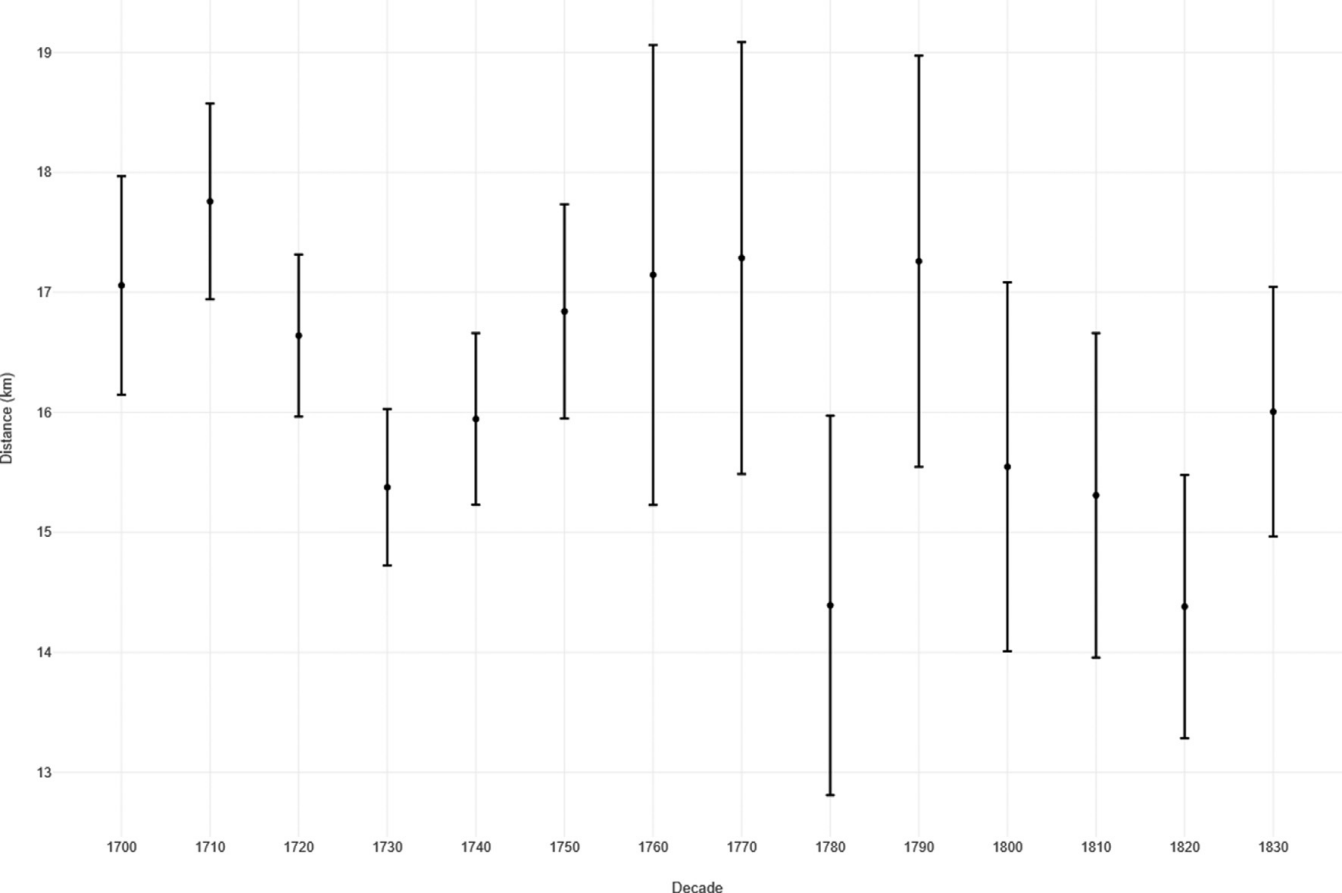
Error plot of distance from home parish to marriage parish, sample of 484 Lincolnshire parishes, 1700–1836. Source: Lincolnshire marriage indexes. Note: Based on N = 14,243 (where distance between co-ordinates is > 0). NB 1830 covers the period 1830-36. Outliers omitted. Bars showing 95% C.I.

Figure 5 represents a boxplot of the distributions of distances in kilometres from the ‘home parish’ to the ‘marriage parish’ for grooms and brides combined for all events where the distance is greater than zero. The median distances range from 6.4km in 1780 to 12.1km in 1700. The bounds of the boxes (at Q1 and Q3) remain relatively constant throughout the period (Q1 range = 3.0km–6.3km; Q3 range = 18.4km–26.5km). In all decades, there are clearly a number of cases where the bride or groom travelled relatively long distances between their ‘home’ and ‘marriage’ parishes. Indeed, these figures are ‘as the crow flies’ so, as such, the real travelling distance may well be significantly greater. To help contextualise these figures, the distances between Stamford and Barton-upon-Humber - the most southerly and northerly major towns in the historical county of Lincolnshire - is 114km as the crow flies, and 141km via the modern road system [60]. Figure 6 represents the mean distance between ‘home’ and ‘marriage’ parish, again for events where the distance is greater than zero. However, here we see that the overlapping error bars (at 95% confidence interval) suggests that the level of change over time is not significant.

**Figure 7:**
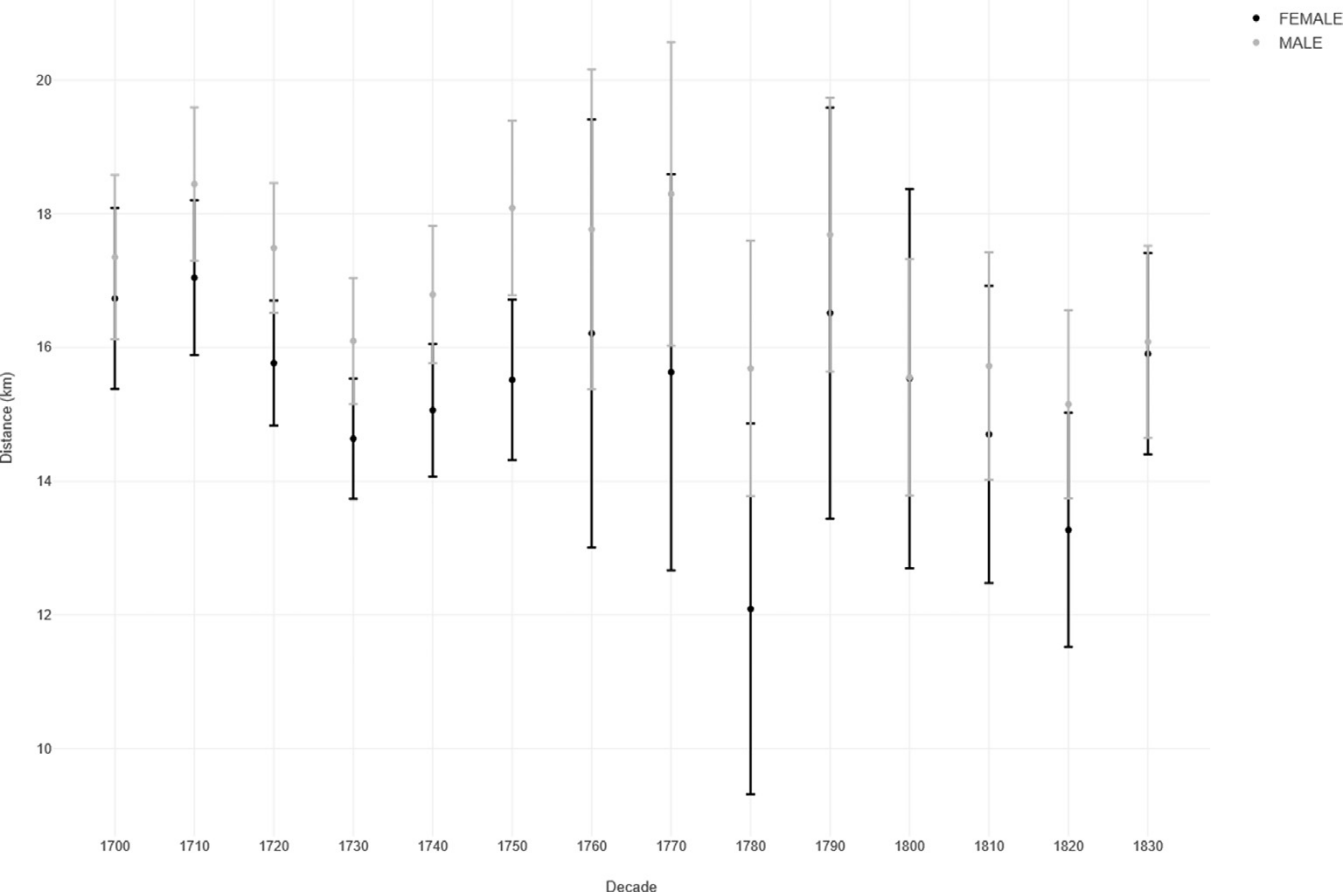
Error plot of distance from home parish to marriage parish, by gender, sample of 484 Lincolnshire parishes, 1700–1836. Source: Lincolnshire marriage indexes. Note: Based on N = 14,243 (where distance between co-ordinates is > 0). NB 1830 covers the period 1830-36. Outliers omitted. Bars showing 95% C.I.

Figure 7 breaks down the distances by gender, again with error bars at 95% C.I. In almost all decades we see that males travel further than females, at least as measured by the mean distance between ‘home’ and ‘marriage’ parish. However, for all but one decade, the error bars overlap, suggesting that the difference between the two genders is not significant. This is contrary to the classic interpretation of gender and distance as espoused by Ravenstein [15].

### 3.3 Inter-regional migration

Beyond the distances between parishes as a crude indicator of migration (for marriage), we can also explore whether there are any patterns of travel within the county. Using chord diagrams, we are able to explore the patterns of travel between ‘home’ and ‘marriage’ parishes using various administrative boundaries. The most important jurisdictional boundaries for the population in the eighteenth- and nineteenth-centuries would be the national and parish level. However, considering that 629 ‘home’ parishes' and 496 ‘marriage’ parishes this would give 311,984 possible combinations. As noted in the Introduction and Materials and Methods sections, Lincolnshire is divided into ‘Ridings’ (with one, Lindsey, in turn divided into three areas) and a series of wapentakes (similar to hundreds in other counties) and boroughs. We may refer to the former as ‘regions’ of the county, and the latter as ‘districts’. Of course, these boundaries do not have any significance in terms of day-to-day life. However, they do serve as a helpful means of exploring patterns of both intra- and inter- region and district migration in Lincolnshire.

**Figure 8:**
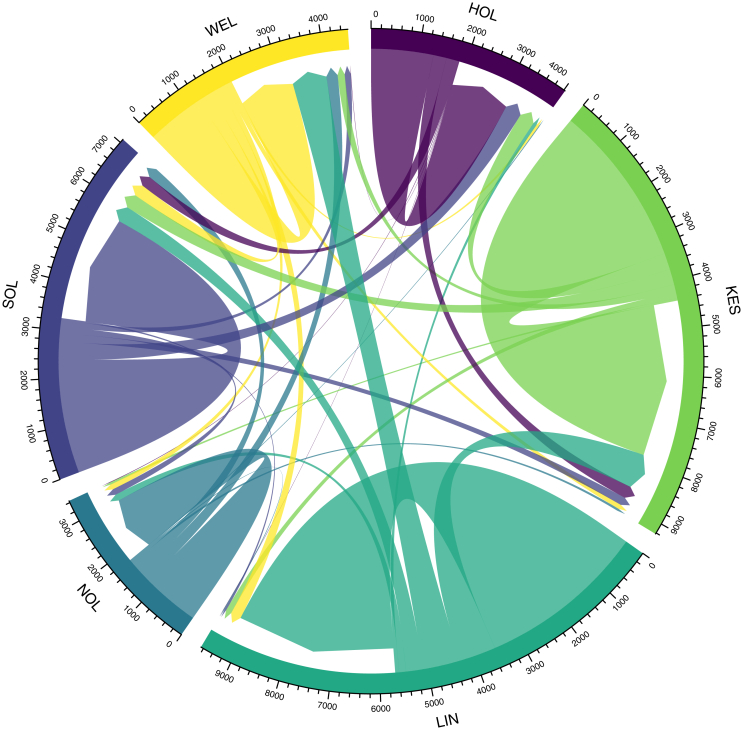
Intra- and inter-regional migration between ‘home’ and ‘marriage’ parishes, 496 Lincolnshire parishes, 1700–1836. Source: Lincolnshire marriage indexes. Notes: HOL = Holland (inc. Boston); KES = Kesteven (inc. Stamford); LIN = Lincoln; NOL = North Lindsey (inc. Great Grimsby); SOL = South Lindsey (inc. Louth); WEL = West Lindsey. N = 18,979.

Figure 8 shows the movement of people from the ‘home’ to ‘marriage’ parish both within and between regions for the entire dataset. The numbers around the axis represent the total number of events, while the initials represent the regions. The arrows represent the movement of people from ‘home’ to ‘marriage’ parish. We can immediately make a number of observations using this chord diagram. Firstly, with the exception of the Holland region, the majority of migration is occurring *within* regions. We can see this is especially the case in Lincoln - something which we will explore in more depth shortly. In North Lindsay, almost all of the migration is occurring within the region. This may be related to the relatively isolated nature of the region in the far north-east of the county. In Kesteven, South Lindsey and West Lindsey, ‘internal’ migration accounts for more than three-quarters of all migration. Given our findings about the relatively short distances which the majority of migrants traveled between their ‘home’ and ‘marriage’ parishes, this should not come as a surprise.

What is perhaps immediately striking is that there is relatively little marriage migration *into* Lincoln. Although Lincoln's *intra-*city migration will be covered shortly, we might observe that migration *from* Lincoln - predominantly into Kesteven, West and South Lindsey - greatly exceeds immigration.

### 3.4 Intra-regional migration

We can next move to explore intra-regional migration. Before doing so, we must begin with a note of caution. The data are neither a complete set of all parishes or a representative sample; furthermore, in some cases the wapentakes are characterised by very total numbers of figures. These figures, then, should be seen as ‘indicative’ only and may well be misleading. As such, no attempt is made to try to *explain* these figures as they may well be erroneous. However, they are presented as they show the potential of this kind of research when employed on a full county dataset.

**Figure 9:**
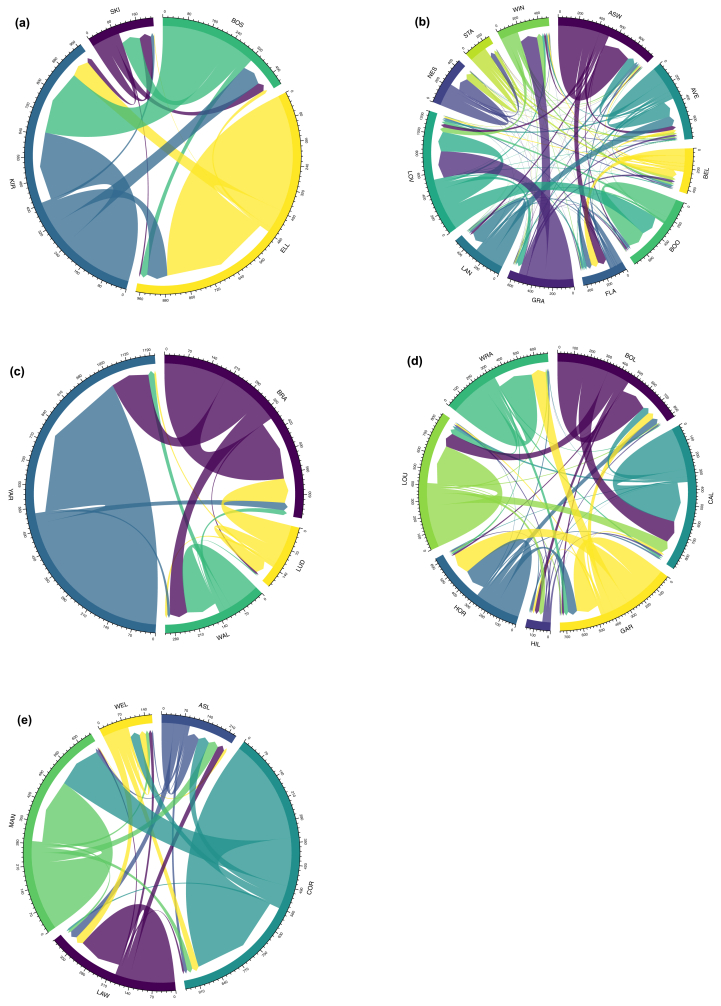
Intra-regional migration between ‘home’ and ‘marriage’ parishes, (a) Holland, (b) Kesteven, (c) North Lindsey; (d) South Lindsey; (e) West Lindsey, 1700–1836. Source: Lincolnshire marriage indexes. Notes: For wapentake/borough abbreviations, see Figure 8.

Figure 9 (a-e) shows the intra-regional migration for the five 'regions' of Lincolnshire. In almost all cases we see, perhaps as per expectations given the short distances of marriage migration, a majority of migration occurs *within* the 'region'. A further preliminary observation is that the major towns in each region appear, in common with Lincoln, to be as much *senders* of migrants as receivers. This is the case with Boston in Holland (Figure 9-a); Louth in North Lindsey (Figure 9-d); Grantham in Kesteven (Figure 9-b). On the other hand, Horncastle does appear to be a more significant receiver rather than sender (Figure 9-d).

### 3.5 Intra-district migration

**Figure 10:**
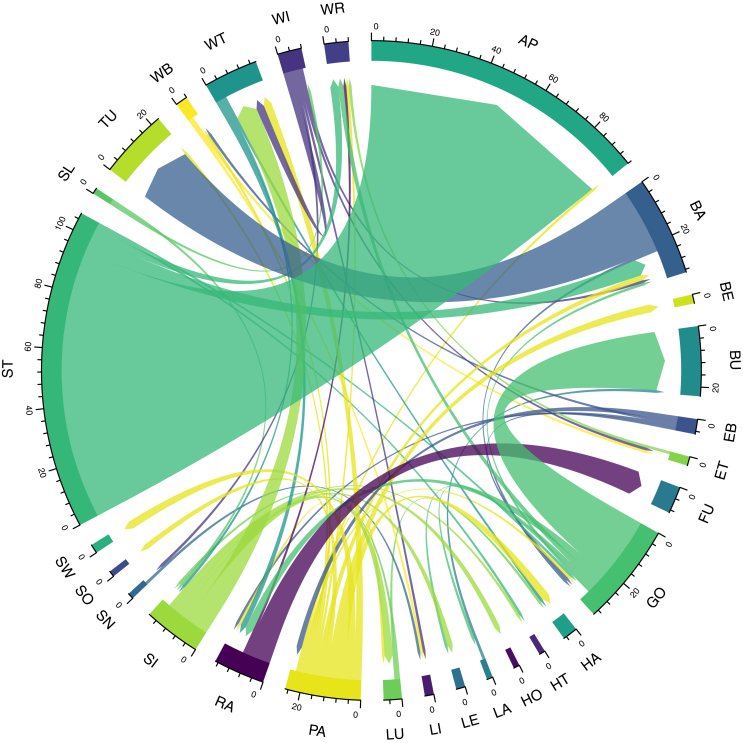
Intra-district migration between the parishes of the wapentake of Wraggoe (Lincolnshire), 1700–1836. Source: Lincolnshire marriage indexes. Notes: AP = Apley; BA = Bardney; BE = Benniworth; BU = Bullington; EB = East Barkwith; ET = East Torrington; FU = Fulnetby; GO = Goltho; HA = Hainton; HT = Hatton; HO = Holton cum Beckering; LA = Langton by Wragby; LE = Legsby; LI = Lissington; LU = Ludford Magna with Ludford Parva; PA = Panton; RA = Rand; SI = Sixhills; SN = Snelland; SO = Sotby; SW = South Willingham; ST = Stainfield; SL = Stainton by Langworth; TU = Tupholme; WB = West Barkwith; WT = West Torrington; WI = Wickenby; WR = Wragby.

It is also possible to explore micro-geographies of migration using this dataset. Space prevents a complete exploration of internal migration within each district in this sample; but for the purposes of demonstrating the potential of utilising large-scale marriage register datasets, one wapentake is explored in this section. Wraggoe is a wapentake with a medium ranked population in the county. It is primarily selected because our sample matches 100% to the figures given in the PRA. The wapentake consists of 28 parishes as covered by our sample in terms of either sending or receiving parishes. The wapentake is located in South Lindsey in the primarily agricultural Lincolnshire Wolds. Figure 10 shows the intra-district migration from ‘home’ to ‘marriage’ parish for the wapentake of Wraggoe. Although the numbers are often very small, we see a number of patterns emerging. Some parishes are almost uniformly *senders* - such as Sixhills (SI) and Goltho (GO) - while others are almost uniformly *receivers* - such as Bullington (BU) and Fulnetby (FU). Again, however, we need to be aware of the ecclesistical micro-histories of the area and administrative idiosyncrasies to avoid misinterpretation. While technically separate parishes, the parish church at Apley serviced adjoining Stainfield for many events. As such, we are not seeing migration as such, but rather an ‘overspill’ of marriages occurring in Apley. Bullington (BU) adjoins Goltho (GO) with the former encompassing the latter after the period under observation, again showing the close geographical and administrative relationship between the two. Bardney (BA) and Tupholme (TU) also adjoin each other.

### 3.6 Migration within the city of Lincoln

As noted above, the foregoing section was based upon the analysis of distances greater than zero. In a further 3,600 cases, however, the distance between parishes was classified as zero. This is, in fact, a by-product of the measuring system which considers parishes within one town or city as having the same coordinates. Of these, 3,544 occurred within the city of Lincoln (plus 33 in different parishes in Stamford, 13 in Wainfleet and the remainder in other towns). Again, with a coverage of 81.2% for the three sample years in the early nineteenth-century, the sample for Lincoln is not complete. As such, we do not elaborate reasons for the possible movement within the city.

**Figure 11:**
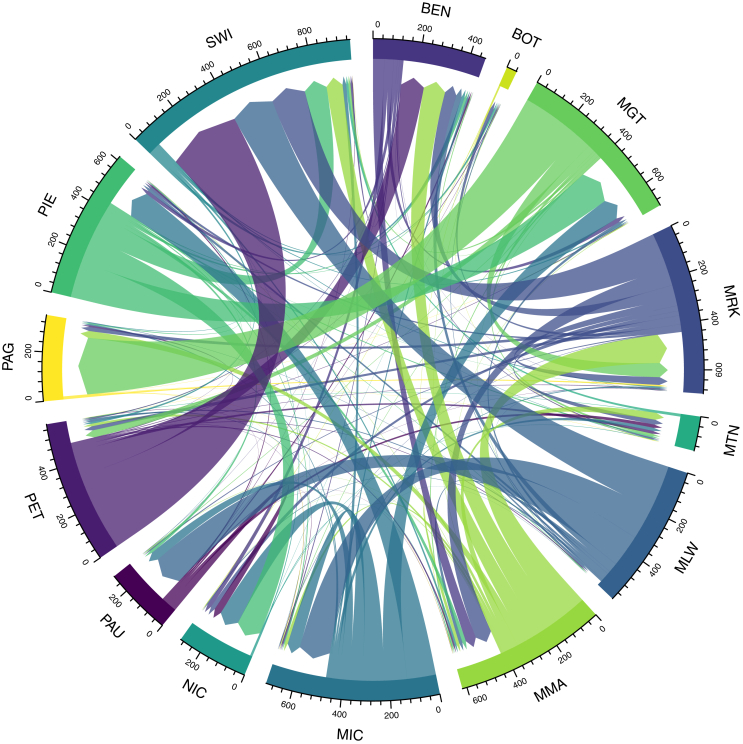
‘Migration’ between ‘home’ and ‘marriage’ parishes, city of Lincoln, 1700–1836. Source: Lincolnshire marriage indexes. Notes: St Benedict (BEN); St Botolph (BOT); St Margaret in the Close (MGT); St Mark (MRK); St Martin (MRT); St Mary le Wigford (MLW); St Mary Magdalen in the Bail (MMA); St Michael on the Mount (MIC); St Nicholas with St John (NIC); St Paul in the Bail (PAU); St Peter at Arches (PET); St Peter at Gowts (PAG); St Peter in Eastgate (PIE); St Swithin (SWI).

Figure 11 shows the ‘home’ and ‘marriage’ parish for Lincoln city between 1700 and 1836. Note that where these are the same they are not presented here. We immediately see a tremendous criss-cross of migration for marriage across the city. The second is that, in common with the analysis of Wraggoe above, some parishes (such as St Botolph (BOT), St Martin (MRT)), St Peter at Gowts (PAG)) are almost exclusively *sending* parishes; while others (such as and St Swithins (SWT) and St Nicholas with St John (NIC)) are almost exclusively *receiving*. A third set of parishes, meanwhile, have a greater balance between sending and receiving (e.g. St Margaret-in-the-Close (MGT) and St Michael-on-the- Mount (MIC)). In order to understand these patterns, it is necessary to undertake a micro-history of the ecclesiastical and social history of Lincoln which is beyond the scope of this study [72]. Such relevant factors may be related to the *status, size* and/or location of the church. St. Botolphs, for example, was referred to ‘second only to the cathedral’ [62]. Other, more unique elements could be at play too. The churach of St Peter at Arches was demolished in the early eighteenth-century and rebuilt; inevitably having an effect on generating more ‘sending’ marriage migrants. This may well be related to simple proximity. St Swithins was, indeed, the closest church to St Peter-at-Arches. Finally, there may well be administrative reasons to choose to marry in one church over another.

## 4 Discussion

Before discussing the possible implications and uses of these findings, it is critical to first explore the limitations of both the sample and the study as a whole. Firstly, we are not using a complete dataset of parishes for the whole county; nor can we be certain that this is a representative sample. Although there does not appear to be a very significant temporal or spatial skewing of the data, coverage is inevitably uneven across the county. This means that any interpretation of such data must be highly provisional, given that it is based upon incomplete data which may present a misleading view of the complete picture. This is linked to a further limitation of the study which is that we have not adequately explored *why* certain migration patterns occur. As mentioned, this is primarily due to the incomplete nature of the dataset. However, the exploration of the micro-level migration within the wapentake of Wraggoe and within the city of Lincoln not only show how a variety of practical, spatial and administrative factors could be both shaping flows of migration; but also suggests that such ‘migration’ may not even be migration at all, but rather simply the performance of an administrative rite in an adjoining parish. While local histories may shed light on some of these issues, we are unlikely to be able to truly understand the reasons for much of this migration, or to disentangle the difference between real migration and choosing to marry in another parish for other reasons.

A second major limitation is that we focus on regions and districts as units of analysis which, in practice, would have meant very little to the population in our sample. Movement across wapentakes to neighbouring parishes has not been explored in this study, but it is highly likely to be a major feature of the movement between ‘home’ and ‘marriage’ parishes. Again, however, this approach was taken for the purposes of ease of presentation. Meanwhile, Van Lottum [8], for example, has identified the development of distinct ‘macro-regions’ which were supplying labour to key industrial centres during this period. As such, the historic county may not be the correct unit of analysis if the hinterland of such an industrial centre straddles one or more historical county boundaries. In the case of Lincolnshire, for example, the rapidly growing town of Kingston-upon-Hull lies just over the River Humber which marks the northern boundary of the county. An alternative analysis might place Kingston-upon-Hull at the centre, and northern Lincolnshire and the East Riding of Yorkshire as the hinterland. However, in this paper, data constraints mean that this is not possible.

A third limitation of the study is that we use indexes of marriages rather than the actual marriage registers themselves. Had the latter been used, we would have been able to perform more complex statistical analysis by exploring patterns of migration by age, or even occupation. However, while such detail can be enlightening, we would quickly run into the ‘small number problem’ at the local level.

A fourth limitation of the study is that we only explore domestic migration within the county of Lincolnshire. In common with the issue raised in the second limitation above, the county boundary would, in practice, have meant very little to the majority of the population in our study here. Indeed, the majority of ‘home parishes’ which are outside of Lincolnshire in this study can be found in neighbouring counties; and, of those, the majority are in parishes either bordering Lincolnshire, or close to the border. Again, we would expect to see a high degree of marriages occurring across county boundaries, but where parishes may well be contiguous. Finally, without exploring marriage data from other counties, we are not able to examine *out-migration* from Lincolnshire to other counties. This could be especially important in the case of developing urban centres such as Kingston-upon-Hull which are located close to the Lincolnshire county boundaries.

Taking these limitations together, then, we move to interpret the results here in only a provisional manner.

A first contribution of the study is to identify the fact that the vast majority of first marriages (around 95%) occur between couples whose ‘home’ parish and ‘marriage’ are one and the same; inother words that no migration (either real or ‘administrative’) occurs. Again, returning to the discussion of the value of using marriage registers as a means of studying migration, the argument by Smith et al. [44] that they represent a significant under-representation of overall migration is well noted and can well be linked to our study. However, it is important to specify that finding *total* patterns of migration is not the aim of this study; rather to explore the patterns of a very particular type of migration, namely migration before marriage (or migration *to* marry). This kind of migration would normally occur at a young age and may well, indeed, be followed by multiple migration events.

Taken together, then, the evidence from Lincolnshire suggests that only a small percentage of the population migrated before marriage (around 5%). These figures, however, are notably larger than found in other studies - for example the study of Oxfordshire discussed in the introduction [21]. Without further systematic analysis of the registers themselves we are not able to conclude whether Lincolnshire was more ‘local’ than Oxfordshire during the 18th century, or whether there is some other explanation, perhaps relating to the recording of information in the parish registers. Yet, when combined with the small distances which marriages which *are* contracted across parishes, we may well state that the ‘relative immobility of the population due to the comparatively stable rural economy’ referred to by Parker [23] appears to be a clear part of reality as reflected through migration - at least *before* marriage in Lincolnshire. These short distances accord with the general literature discussed in the introduction [22]. Only further analysis both on Lincolnshire, and in comparative context, will show whether the county was truly more ‘local’ than other places and, in turn, how this (lack of) geographical and social mobility might be linked to other feature of economy and society, such as social customs [25]. In other words, we can only provisionally state that our two hypotheses as set out earlier in the study are proven correct.

The analysis of inter- and intra- regional and district migration revealed some interesting glimpses - albeit with the strong caveats set out above. Firstly, as anticipated by other studies of eighteenth- and early nineteenth-century migration in England (and elsewhere) [11, 63], there does not appear to be one set ‘model’ of migration - contrary to the stereotype of an influx of people from rural to large urban centres. Of course, Lincolnshire was a rural county relatively little affected by the Industrial Revolution during this period [64]; and even its largest towns and cities were relatively modest in size compared to other counties. Despite this, there is little evidence that a simple model of movement into towns was a motivation for young, pre-marriage migrants *within* Lincolnshire. Again, though, without information on motivation, or a more in-depth analysis of the characteristics of both migrants and their sending/receiving communities it is not possible to contribute to the discussion by Hollingsworth in the Introduction concerning the primacy of economic over social motivation to migrate longer distances [22], to the potential role of the Poor Law e.g. [6] - or indeed, to other theories of migration [15, 65, 66]. We can also accord with Pooley and Turbull [11] - *contra* Ravenstein [15] - that for pre-marriage migration at least, male migration does not appear to be over greater distances than female; and that urban and rural populations appear equally susceptible to moving.

The examples from Wraggoe and Lincoln show the importance of exploring micro-historical contexts in order to understand the patterns of migration. Crucially, these also provide an important methodological contribution. Many demographic studies which have concentrated on single parishes (or small groups of parishes without considering their wider context) have been criticised for not adequately taking into account either migration or, in the case of births and deaths, non-Anglican registration [37, 67]. These issues can be especially pertinent in urban and/or industrial locations [36, 37, 68, 69]. The evidence presented here clearly demonstrates how a concentration on one parish alone - especially in an urban multi-parish context - not only presents a skewed picture of the parish itself, but also ignores an essential, and important feature of demographic activity, namely migration in and out of the parish under study.

The next step in the analysis of marriage migration in England through the approach taken in this paper, would be to explore the extent to which the findings in this paper are generalisable to counties and regions with other social and economic characteristics. In this paper, our analysis is solely confined to Lincolnshire - an agricultural county with a handful of urban centres which are, in effect market towns. While the county was often at the forefront of agricultural technology, we are exploring a social and economic world which is rather insulated from many of the aspects of urbanisation, and economic and social transformation which we generally associate with the English Industrial Revolution. As Van Lottum demonstrated [8], industrialisation led to a shrinking of migration fields over time (and a simultaneous emergence of quite distinct labour supplying 'macro' regions for the key industrial centres of the modern age). This, in turn, suggests a dynamism in migratory behaviour which could map onto the general findings and interpretation of our study here. Expanding this mode of analysis, therefore, to other geographical typologies - such as industrial regions, or regions with a large urban centre and more obvious hinterland - is a logical next step to build up a more holistic understanding of the general characteristics of migration in the long eighteenth-century.

## 5 Conclusion

This study takes a novel approach to studying some of the most hidden aspects of (British) historical demography, namely internal migration in the pre-census era. Not only is a novel, large dataset employed; but it is also perhaps the first time that chord diagrams (circular visualisation) has been used to show patterns of inter- and intra-regional/district migration for the pre-Census era in the UK. As such, the study not only contributes to the empirical and theoretical understanding of historical migration, but also points to key possible avenues of further study utilising large, digital datasets and new modes of data processing and visualisation.

This study has contributed to the broader study of migration in eighteenth- and nineteenth-century England in a variety of ways. It has demonstrated the predominantly local character of both the contracting of marriage as well as migration before/for marriage. On the other hand, it has demonstrated that inter- and intra- regional and district migration was also a non-trivial component of population movement during this time. The study has applied novel methods of visualising historical migration data and explored the importance of understanding the micro-historical context of geographies, societies, economy and administration in properly interpreting historical migration data.

However, a primary goal of this study is to demonstrate the potential use of using large marriage register datasets. Migration is, by definition, a multi-dimensional process. The matrix of flows becomes more realistic by adding ever more data points. Furthermore, our units of analysis within which recorded migration occurs - be they counties, regions, districts or otherwise - often do not map onto the real lived experiences of the populations under study. By studying migration at the county level for Lincolnshire - and by performing relatively simple geospatial analysis and deploying novel data visualisation techniques - we have been able to present a snapshot of a particular process of migration which was previously hidden in the historical record.

The next logical step is to delve more *deeply* into the local context, by exploring more information as derived transcribing full marriage registers; but then also to *broaden* the scope of analysis in order to present Lincolnshire in a comparative context, but also to explore inter-county, longer-distance migration.

## Declarations

### Author contribution statement

Stuart Gietel-Basten: Conceived and designed the experiments; Performed the experiments; Analyzed and interpreted the data; Contributed reagents, materials, analysis tools or data; Wrote the paper.

### Funding statement

This research did not receive any specific grant from funding agencies in the public, commercial, or not-for-profit sectors.

### Data availability statement

Data included in supplementary material.

### Declaration of interests statement

The authors declare no conflict of interest.

### Additional information

No additional information is available for this paper.

## Footnotes

1 Gietel-Basten, S.A., 2020, "Replication Data for: chord diagrams used in analysis of intra-county migration", https://doi.org/10.14711/dataset/RNKQRI, DataSpace@HKUST, V1
