# Supplementary material for: Circular visualisation of historical migration in England in the long eighteenth-century
Source: Heliyon. 2020 Nov 19;6(11):e05490. doi: 10.1016/j.heliyon.2020.e05490 (PMC7689171; doi:10.1016/j.heliyon.2020.e05490)
Supplement: Appendix 1 [file mmc1.docx]

**Appendix 1: Number of marriages per parish, 1700-1837**

| **Region** | **Wapentake/borough** | **Parish** | **N** |
| --- | --- | --- | --- |
| Holland | Boston | Boston | 7088 |
| Holland | Elloe | Cowbit | 417 |
| Holland | Elloe | Crowland | 1655 |
| Holland | Elloe | Fleet | 619 |
| Holland | Elloe | Gedney | 988 |
| Holland | Elloe | Gedney Hill | 350 |
| Holland | Elloe | Holbeach | 2730 |
| Holland | Elloe | Moulton | 1378 |
| Holland | Elloe | Pinchbeck | 2417 |
| Holland | Elloe | Spalding | 4726 |
| Holland | Elloe | Sutton St Edmund | 2119 |
| Holland | Elloe | Sutton St James | 259 |
| Holland | Elloe | Sutton St Nicholas | 431 |
| Holland | Elloe | Tydd St Mary | 679 |
| Holland | Elloe | Weston | 469 |
| Holland | Elloe | Whaplode | 1800 |
| Holland | Kirton | Algarkirk | 642 |
| Holland | Kirton | Bicker | 745 |
| Holland | Kirton | Brothertoft | 243 |
| Holland | Kirton | Donington | 1362 |
| Holland | Kirton | Fosdyke | 202 |
| Holland | Kirton | Frampton | 657 |
| Holland | Kirton | Gosberton | 1313 |
| Holland | Kirton | Kirton in Holland | 1303 |
| Holland | Kirton | Quadring | 613 |
| Holland | Kirton | Surfleet | 699 |
| Holland | Kirton | Sutterton | 562 |
| Holland | Kirton | Swineshead | 1614 |
| Holland | Kirton | The Friths | 48 |
| Holland | Kirton | Wigtoft | 662 |
| Holland | Kirton | Wyberton | 379 |
| Holland | Skirbeck | Benington | 536 |
| Holland | Skirbeck | Butterwick | 193 |
| Holland | Skirbeck | Fishtoft | 332 |
| Holland | Skirbeck | Freiston | 723 |
| Holland | Skirbeck | Leake | 809 |
| Holland | Skirbeck | Leverton | 379 |
| Holland | Skirbeck | Skirbeck | 1165 |
| Holland | Skirbeck | Wrangle | 672 |
| Kesteven | Aswardhurn | Aunsby | 76 |
| Kesteven | Aswardhurn | Burton Penwardine | 103 |
| Kesteven | Aswardhurn | Evedon | 146 |
| Kesteven | Aswardhurn | Ewerby | 344 |
| Kesteven | Aswardhurn | Great Hale | 854 |
| Kesteven | Aswardhurn | Heckington | 982 |
| Kesteven | Aswardhurn | Helpringham | 492 |
| Kesteven | Aswardhurn | Heydour | 603 |
| Kesteven | Aswardhurn | Howell | 247 |
| Kesteven | Aswardhurn | Ingoldsby | 226 |
| Kesteven | Aswardhurn | Kirkby Laythorpe with Asgarby | 183 |
| Kesteven | Aswardhurn | Quarrington | 376 |
| Kesteven | Aswardhurn | Scredington | 203 |
| Kesteven | Aswardhurn | Silk Willoughby | 309 |
| Kesteven | Aswardhurn | South Kyme and North Kyme | 536 |
| Kesteven | Aveland | Aslackby | 351 |
| Kesteven | Aveland | Billingborough | 687 |
| Kesteven | Aveland | Bourne | 2112 |
| Kesteven | Aveland | Brauncewell with Dunsby | 68 |
| Kesteven | Aveland | Dembleby | 55 |
| Kesteven | Aveland | Dowsby | 164 |
| Kesteven | Aveland | Folkingham with Laughton | 945 |
| Kesteven | Aveland | Haceby | 112 |
| Kesteven | Aveland | Horbling | 466 |
| Kesteven | Aveland | Kirkby Underwood | 226 |
| Kesteven | Aveland | Morton with Hacconby | 922 |
| Kesteven | Aveland | Newton | 148 |
| Kesteven | Aveland | Osbournby | 343 |
| Kesteven | Aveland | Pickworth | 162 |
| Kesteven | Aveland | Rippingale | 429 |
| Kesteven | Aveland | Scott Willoughby | 28 |
| Kesteven | Aveland | Sempringham with Pointon and Birthorpe | 498 |
| Kesteven | Aveland | Spanby | 94 |
| Kesteven | Aveland | Swaton | 297 |
| Kesteven | Aveland | Threckingham | 187 |
| Kesteven | Beltisloe | Bassingthorpe with Westby | 122 |
| Kesteven | Beltisloe | Bitchfield | 136 |
| Kesteven | Beltisloe | Burton le Coggles | 188 |
| Kesteven | Beltisloe | Castle Bytham | 333 |
| Kesteven | Beltisloe | Corby | 339 |
| Kesteven | Beltisloe | Creeton with Counthorpe | 79 |
| Kesteven | Beltisloe | Edenham | 469 |
| Kesteven | Beltisloe | Holywell | 75 |
| Kesteven | Beltisloe | Irnham | 414 |
| Kesteven | Beltisloe | Lenton | 258 |
| Kesteven | Beltisloe | Little Bytham | 207 |
| Kesteven | Beltisloe | North Witham | 162 |
| Kesteven | Beltisloe | Skillington | 293 |
| Kesteven | Beltisloe | South Witham | 328 |
| Kesteven | Beltisloe | Stainby with Gunby [St Nicholas] | 124 |
| Kesteven | Beltisloe | Swayfield | 171 |
| Kesteven | Beltisloe | Swinstead | 197 |
| Kesteven | Beltisloe | Witham on the Hill | 453 |
| Kesteven | Boothby Graffoe | Aubourn | 194 |
| Kesteven | Boothby Graffoe | Bassingham | 358 |
| Kesteven | Boothby Graffoe | Boothby Graffoe | 143 |
| Kesteven | Boothby Graffoe | Boultham | 322 |
| Kesteven | Boothby Graffoe | Bracebridge | 257 |
| Kesteven | Boothby Graffoe | Carlton le Moorland | 393 |
| Kesteven | Boothby Graffoe | Coleby | 260 |
| Kesteven | Boothby Graffoe | Doddington with Whisby | 232 |
| Kesteven | Boothby Graffoe | Eagle | 199 |
| Kesteven | Boothby Graffoe | Harmston | 310 |
| Kesteven | Boothby Graffoe | Navenby | 373 |
| Kesteven | Boothby Graffoe | North Hykeham | 295 |
| Kesteven | Boothby Graffoe | North Scarle | 391 |
| Kesteven | Boothby Graffoe | Norton Disney | 205 |
| Kesteven | Boothby Graffoe | Skellingthorpe | 247 |
| Kesteven | Boothby Graffoe | Skinnand | 5 |
| Kesteven | Boothby Graffoe | Stapleford | 138 |
| Kesteven | Boothby Graffoe | Swinderby | 218 |
| Kesteven | Boothby Graffoe | Thorpe on the Hill | 252 |
| Kesteven | Boothby Graffoe | Thurlby (near Lincoln) | 83 |
| Kesteven | Boothby Graffoe | Waddington | 499 |
| Kesteven | Boothby Graffoe | Welbourn | 422 |
| Kesteven | Boothby Graffoe | Wellingore | 581 |
| Kesteven | Flaxwell | Anwick | 185 |
| Kesteven | Flaxwell | Ashby de la Launde | 347 |
| Kesteven | Flaxwell | Cranwell | 100 |
| Kesteven | Flaxwell | Digby with Bloxholm | 155 |
| Kesteven | Flaxwell | Dorrington | 243 |
| Kesteven | Flaxwell | Leasingham with Roxholme | 285 |
| Kesteven | Flaxwell | New Sleaford | 1779 |
| Kesteven | Flaxwell | Rowston | 208 |
| Kesteven | Flaxwell | Ruskington | 441 |
| Kesteven | Grantham | Grantham | 4290 |
| Kesteven | Langoe | Billinghay | 1059 |
| Kesteven | Langoe | Blankney | 421 |
| Kesteven | Langoe | Branston | 995 |
| Kesteven | Langoe | Canwick | 220 |
| Kesteven | Langoe | Dunston | 284 |
| Kesteven | Langoe | Kirkby Green | 66 |
| Kesteven | Langoe | Metheringham | 427 |
| Kesteven | Langoe | Nocton | 135 |
| Kesteven | Langoe | Potter Hanworth | 300 |
| Kesteven | Langoe | Scopwick | 180 |
| Kesteven | Langoe | Timberland | 752 |
| Kesteven | Langoe | Washingborough | 625 |
| Kesteven | Loveden | Ancaster | 240 |
| Kesteven | Loveden | Barkston | 233 |
| Kesteven | Loveden | Barrowby | 358 |
| Kesteven | Loveden | Beckingham | 374 |
| Kesteven | Loveden | Brant Broughton | 470 |
| Kesteven | Loveden | Caythorpe | 406 |
| Kesteven | Loveden | Claypole | 384 |
| Kesteven | Loveden | Denton | 453 |
| Kesteven | Loveden | East Allington | 127 |
| Kesteven | Loveden | Fenton | 119 |
| Kesteven | Loveden | Foston | 149 |
| Kesteven | Loveden | Fulbeck | 445 |
| Kesteven | Loveden | Great Gonerby | 523 |
| Kesteven | Loveden | Harlaxton | 273 |
| Kesteven | Loveden | Honington | 102 |
| Kesteven | Loveden | Hough on the Hill | 327 |
| Kesteven | Loveden | Hougham | 189 |
| Kesteven | Loveden | Leadenham | 402 |
| Kesteven | Loveden | Long Bennington | 1048 |
| Kesteven | Loveden | Manthorpe with Londonthorpe | 134 |
| Kesteven | Loveden | Marston | 265 |
| Kesteven | Loveden | Normanton | 139 |
| Kesteven | Loveden | Sedgebrook | 203 |
| Kesteven | Loveden | Stragglethorpe | 91 |
| Kesteven | Loveden | Stubton | 137 |
| Kesteven | Loveden | Syston | 116 |
| Kesteven | Loveden | West Allington | 85 |
| Kesteven | Loveden | Westborough | 345 |
| Kesteven | Loveden | Wyville with Hungerton | 10 |
| Kesteven | Ness | Barholme with Stowe | 140 |
| Kesteven | Ness | Baston | 393 |
| Kesteven | Ness | Braceborough | 159 |
| Kesteven | Ness | Carlby | 259 |
| Kesteven | Ness | Deeping St James | 1314 |
| Kesteven | Ness | Greatford | 169 |
| Kesteven | Ness | Langtoft | 393 |
| Kesteven | Ness | Market Deeping | 904 |
| Kesteven | Ness | Tallington | 214 |
| Kesteven | Ness | Thurlby (near Bourne) | 500 |
| Kesteven | Ness | Uffington | 367 |
| Kesteven | Ness | West Deeping | 240 |
| Kesteven | Ness | Wilsthorpe | 42 |
| Kesteven | Stamford | Stamford All Saints with St Peter | 1081 |
| Kesteven | Stamford | Stamford St George with St Paul | 768 |
| Kesteven | Stamford | Stamford St John with St Clement | 615 |
| Kesteven | Stamford | Stamford St Mary | 2219 |
| Kesteven | Stamford | Stamford St Michael with St Andrew and St Stephen | 1224 |
| Kesteven | Winnibriggs and Threo | Boothby Pagnell | 108 |
| Kesteven | Winnibriggs and Threo | Braceby | 62 |
| Kesteven | Winnibriggs and Threo | Colsterworth | 512 |
| Kesteven | Winnibriggs and Threo | Great Ponton | 310 |
| Kesteven | Winnibriggs and Threo | Little Ponton | 394 |
| Kesteven | Winnibriggs and Threo | Ropsley | 351 |
| Kesteven | Winnibriggs and Threo | Sapperton | 52 |
| Kesteven | Winnibriggs and Threo | Stroxton | 104 |
| Kesteven | Winnibriggs and Threo | Welby | 217 |
| Kesteven | Winnibriggs and Threo | Wilsford | 269 |
| Kesteven | Winnibriggs and Threo | Woolsthorpe with Stainworth | 352 |
| Lincoln | Lincoln | Lincoln Cathedral | 209 |
| Lincoln | Lincoln | Lincoln St Benedict | 202 |
| Lincoln | Lincoln | Lincoln St Botolph | 622 |
| Lincoln | Lincoln | Lincoln St Margaret in the Close | 878 |
| Lincoln | Lincoln | Lincoln St Mark | 122 |
| Lincoln | Lincoln | Lincoln St Martin | 2077 |
| Lincoln | Lincoln | Lincoln St Mary le Wigford | 824 |
| Lincoln | Lincoln | Lincoln St Mary Magdalen in the Bail | 1203 |
| Lincoln | Lincoln | Lincoln St Michael on the Mount | 153 |
| Lincoln | Lincoln | Lincoln St Nicholas with St John | 22 |
| Lincoln | Lincoln | Lincoln St Paul in the Bail | 806 |
| Lincoln | Lincoln | Lincoln St Peter at Arches | 977 |
| Lincoln | Lincoln | Lincoln St Peter at Gowts | 149 |
| Lincoln | Lincoln | Lincoln St Peter in Eastgate | 474 |
| Lincoln | Lincoln | Lincoln St Swithin | 914 |
| North Lindsey | Bradley-Haverstoe | Ashby cum Fenby | 131 |
| North Lindsey | Bradley-Haverstoe | Aylesby | 107 |
| North Lindsey | Bradley-Haverstoe | Barnoldby le Beck | 191 |
| North Lindsey | Bradley-Haverstoe | Beelsby | 112 |
| North Lindsey | Bradley-Haverstoe | Bradley | 67 |
| North Lindsey | Bradley-Haverstoe | Cabourne | 103 |
| North Lindsey | Bradley-Haverstoe | Caistor | 875 |
| North Lindsey | Bradley-Haverstoe | Clee | 375 |
| North Lindsey | Bradley-Haverstoe | Cuxwold | 61 |
| North Lindsey | Bradley-Haverstoe | Fulstow | 394 |
| North Lindsey | Bradley-Haverstoe | Grainsby | 122 |
| North Lindsey | Bradley-Haverstoe | Great Coates | 253 |
| North Lindsey | Bradley-Haverstoe | Great Grimsby | 1280 |
| North Lindsey | Bradley-Haverstoe | Hatcliffe | 146 |
| North Lindsey | Bradley-Haverstoe | Healing | 177 |
| North Lindsey | Bradley-Haverstoe | Holton le Clay | 135 |
| North Lindsey | Bradley-Haverstoe | Humberstone | 151 |
| North Lindsey | Bradley-Haverstoe | Irby on Humber | 222 |
| North Lindsey | Bradley-Haverstoe | Laceby | 314 |
| North Lindsey | Bradley-Haverstoe | Little Coates | 54 |
| North Lindsey | Bradley-Haverstoe | Marsh Chapel | 298 |
| North Lindsey | Bradley-Haverstoe | North Thoresby | 318 |
| North Lindsey | Bradley-Haverstoe | Rothwell | 110 |
| North Lindsey | Bradley-Haverstoe | Scartho | 144 |
| North Lindsey | Bradley-Haverstoe | Swallow | 249 |
| North Lindsey | Bradley-Haverstoe | Swinhope | 73 |
| North Lindsey | Bradley-Haverstoe | Tetney | 538 |
| North Lindsey | Bradley-Haverstoe | Waithe | 65 |
| North Lindsey | Bradley-Haverstoe | Waltham | 342 |
| North Lindsey | Bradley-Haverstoe | Wold Newton | 92 |
| North Lindsey | Ludborough | Covenham St Bartholomew | 201 |
| North Lindsey | Ludborough | Covenham St Mary | 116 |
| North Lindsey | Ludborough | Fotherby and Brackenborough | 140 |
| North Lindsey | Ludborough | Little Grimsby | 46 |
| North Lindsey | Ludborough | Ludborough | 430 |
| North Lindsey | Ludborough | North Ormesby | 69 |
| North Lindsey | Ludborough | Utterby | 167 |
| North Lindsey | Ludborough | Wyham with Caldeby | 68 |
| North Lindsey | Walshcroft | Binbrook St Mary and St Gabriel | 421 |
| North Lindsey | Walshcroft | Claxby with Normanby le Wold | 146 |
| North Lindsey | Walshcroft | Croxby | 66 |
| North Lindsey | Walshcroft | Holton le Moor | 13 |
| North Lindsey | Walshcroft | Linwood | 153 |
| North Lindsey | Walshcroft | Market Rasen | 721 |
| North Lindsey | Walshcroft | Middle Rasen | 412 |
| North Lindsey | Walshcroft | Newton by Toft | 75 |
| North Lindsey | Walshcroft | North Willingham | 170 |
| North Lindsey | Walshcroft | Owersby with Kirkby and Osgodby | 195 |
| North Lindsey | Walshcroft | South Kelsey | 472 |
| North Lindsey | Walshcroft | Tealby | 428 |
| North Lindsey | Walshcroft | Thoresway | 75 |
| North Lindsey | Walshcroft | Thorganby | 93 |
| North Lindsey | Walshcroft | Thornton le Moor | 76 |
| North Lindsey | Walshcroft | Usselby | 88 |
| North Lindsey | Walshcroft | Walesby | 158 |
| North Lindsey | Walshcroft | West Rasen | 179 |
| North Lindsey | Yarborough | Barnetby le Wold | 220 |
| North Lindsey | Yarborough | Barrow upon Humber | 794 |
| North Lindsey | Yarborough | Barton on Humber | 1874 |
| North Lindsey | Yarborough | Bigby | 357 |
| North Lindsey | Yarborough | Bonby | 150 |
| North Lindsey | Yarborough | Brocklesby | 137 |
| North Lindsey | Yarborough | Cadney cum Howsham | 178 |
| North Lindsey | Yarborough | Clixby | 10 |
| North Lindsey | Yarborough | Croxton | 50 |
| North Lindsey | Yarborough | East Halton | 423 |
| North Lindsey | Yarborough | Elsham | 365 |
| North Lindsey | Yarborough | Goxhill | 803 |
| North Lindsey | Yarborough | Grasby | 165 |
| North Lindsey | Yarborough | Habrough | 347 |
| North Lindsey | Yarborough | Horkstow | 326 |
| North Lindsey | Yarborough | Immingham | 236 |
| North Lindsey | Yarborough | Keelby | 284 |
| North Lindsey | Yarborough | Killingholme | 366 |
| North Lindsey | Yarborough | Kirmington | 158 |
| North Lindsey | Yarborough | Limber Magna | 295 |
| North Lindsey | Yarborough | Melton Ross | 87 |
| North Lindsey | Yarborough | Nettleton | 334 |
| North Lindsey | Yarborough | North Kelsey | 353 |
| North Lindsey | Yarborough | Riby | 102 |
| North Lindsey | Yarborough | Saxby All Saints | 315 |
| North Lindsey | Yarborough | Searby cum Ownby | 308 |
| North Lindsey | Yarborough | Somerby | 308 |
| North Lindsey | Yarborough | South Ferriby | 270 |
| North Lindsey | Yarborough | Stallingborough | 331 |
| North Lindsey | Yarborough | Thornton Curtis | 418 |
| North Lindsey | Yarborough | Ulceby | 537 |
| North Lindsey | Yarborough | Wootton | 327 |
| North Lindsey | Yarborough | Wrawby | 1578 |
| South Lindsey | Bolingbroke | Carrington | 372 |
| South Lindsey | Bolingbroke | East Keal | 278 |
| South Lindsey | Bolingbroke | East Kirkby | 224 |
| South Lindsey | Bolingbroke | Hagnaby | 62 |
| South Lindsey | Bolingbroke | Halton Holegate | 598 |
| South Lindsey | Bolingbroke | Hundleby | 224 |
| South Lindsey | Bolingbroke | Little Steeping | 190 |
| South Lindsey | Bolingbroke | Lusby | 244 |
| South Lindsey | Bolingbroke | Mavis Enderby | 154 |
| South Lindsey | Bolingbroke | Midville | 64 |
| South Lindsey | Bolingbroke | Miningsby | 74 |
| South Lindsey | Bolingbroke | Raithby | 535 |
| South Lindsey | Bolingbroke | Revesby | 284 |
| South Lindsey | Bolingbroke | Sibsey | 934 |
| South Lindsey | Bolingbroke | Spilsby | 635 |
| South Lindsey | Bolingbroke | Stickford | 205 |
| South Lindsey | Bolingbroke | Stickney | 495 |
| South Lindsey | Bolingbroke | Thorpe St Peter | 293 |
| South Lindsey | Bolingbroke | Toynton All Saints with Toynton St Peter | 436 |
| South Lindsey | Bolingbroke | West Keal | 480 |
| South Lindsey | Calceworth | Aby and Greenfield, Belleau with Claythorpe | 220 |
| South Lindsey | Calceworth | Alford | 840 |
| South Lindsey | Calceworth | Anderby | 212 |
| South Lindsey | Calceworth | Beesby in the Marsh | 104 |
| South Lindsey | Calceworth | Bilsby | 438 |
| South Lindsey | Calceworth | Claxby | 123 |
| South Lindsey | Calceworth | Cumberworth | 111 |
| South Lindsey | Calceworth | Farlesthorpe | 103 |
| South Lindsey | Calceworth | Gayton le Marsh | 244 |
| South Lindsey | Calceworth | Hannah cum Hagnaby and Markby | 61 |
| South Lindsey | Calceworth | Haugh | 12 |
| South Lindsey | Calceworth | Hogsthorpe | 338 |
| South Lindsey | Calceworth | Huttoft | 234 |
| South Lindsey | Calceworth | Legbourne | 217 |
| South Lindsey | Calceworth | Little Cawthorpe | 82 |
| South Lindsey | Calceworth | Mablethorpe with Stane | 135 |
| South Lindsey | Calceworth | Mumby | 472 |
| South Lindsey | Calceworth | Rigsby with Ailby | 97 |
| South Lindsey | Calceworth | Saleby with Thoresthorpe | 180 |
| South Lindsey | Calceworth | South Reston | 57 |
| South Lindsey | Calceworth | South Thoresby | 187 |
| South Lindsey | Calceworth | Strubby with Woodthorpe | 172 |
| South Lindsey | Calceworth | Sutton le Marsh | 66 |
| South Lindsey | Calceworth | Swaby | 286 |
| South Lindsey | Calceworth | Theddlethorpe All Saints | 6 |
| South Lindsey | Calceworth | Theddlethorpe St Helen with Mablethorpe St Peter | 235 |
| South Lindsey | Calceworth | Tothill | 59 |
| South Lindsey | Calceworth | Trusthorpe | 241 |
| South Lindsey | Calceworth | Well | 405 |
| South Lindsey | Calceworth | Willoughby | 566 |
| South Lindsey | Candleshoe | Addlethorpe | 180 |
| South Lindsey | Candleshoe | Ashby by Partney | 97 |
| South Lindsey | Candleshoe | Bratoft | 120 |
| South Lindsey | Candleshoe | Burgh le Marsh | 617 |
| South Lindsey | Candleshoe | Candlesby | 125 |
| South Lindsey | Candleshoe | Croft | 415 |
| South Lindsey | Candleshoe | Dalby | 74 |
| South Lindsey | Candleshoe | Firsby | 145 |
| South Lindsey | Candleshoe | Friskney | 652 |
| South Lindsey | Candleshoe | Great Steeping | 214 |
| South Lindsey | Candleshoe | Gunby [St Peter] | 113 |
| South Lindsey | Candleshoe | Ingoldmells | 78 |
| South Lindsey | Candleshoe | Irby in the Marsh | 75 |
| South Lindsey | Candleshoe | Orby | 206 |
| South Lindsey | Candleshoe | Partney | 196 |
| South Lindsey | Candleshoe | Scremby | 141 |
| South Lindsey | Candleshoe | Skegness | 130 |
| South Lindsey | Candleshoe | Skendleby | 163 |
| South Lindsey | Candleshoe | Sutterby | 31 |
| South Lindsey | Candleshoe | Wainfleet All Saints | 590 |
| South Lindsey | Candleshoe | Wainfleet St Mary | 329 |
| South Lindsey | Candleshoe | Welton le Marsh | 243 |
| South Lindsey | Candleshoe | Winthorpe | 193 |
| South Lindsey | Gartree | Asterby | 100 |
| South Lindsey | Gartree | Baumber | 243 |
| South Lindsey | Gartree | Belchford | 291 |
| South Lindsey | Gartree | Bucknall | 170 |
| South Lindsey | Gartree | Donington on Bain | 159 |
| South Lindsey | Gartree | Edlington | 167 |
| South Lindsey | Gartree | Gautby | 115 |
| South Lindsey | Gartree | Goulceby | 121 |
| South Lindsey | Gartree | Great Sturton | 101 |
| South Lindsey | Gartree | Hemingby | 180 |
| South Lindsey | Gartree | Horsington | 179 |
| South Lindsey | Gartree | Kirkby on Bain | 560 |
| South Lindsey | Gartree | Kirkstead | 141 |
| South Lindsey | Gartree | Langton | 150 |
| South Lindsey | Gartree | Market Stainton | 91 |
| South Lindsey | Gartree | Martin | 47 |
| South Lindsey | Gartree | Minting | 190 |
| South Lindsey | Gartree | Ranby | 74 |
| South Lindsey | Gartree | Scamblesby | 190 |
| South Lindsey | Gartree | Scrivelsby with Dalderby | 180 |
| South Lindsey | Gartree | Stenigot | 65 |
| South Lindsey | Gartree | Stixwould | 180 |
| South Lindsey | Gartree | Tattershall | 756 |
| South Lindsey | Gartree | Waddingworth | 61 |
| South Lindsey | Gartree | Wispington | 61 |
| South Lindsey | Gartree | Woodhall | 120 |
| South Lindsey | Hill | Ashby Puerorum | 102 |
| South Lindsey | Hill | Aswardby | 215 |
| South Lindsey | Hill | Bag Enderby | 84 |
| South Lindsey | Hill | Brinkhill | 72 |
| South Lindsey | Hill | Claxby Pluckacre | 22 |
| South Lindsey | Hill | Fulletby | 184 |
| South Lindsey | Hill | Greetham | 72 |
| South Lindsey | Hill | Hagworthingham | 305 |
| South Lindsey | Hill | Harrington | 81 |
| South Lindsey | Hill | Langton by Spilsby | 141 |
| South Lindsey | Hill | Oxcombe | 22 |
| South Lindsey | Hill | Salmonby | 108 |
| South Lindsey | Hill | Sausthorpe | 123 |
| South Lindsey | Hill | Somersby | 77 |
| South Lindsey | Hill | South Ormesby with North Ormesby | 234 |
| South Lindsey | Hill | Tetford | 306 |
| South Lindsey | Hill | Winceby | 41 |
| South Lindsey | Horncastle Soke | Coningsby | 1587 |
| South Lindsey | Horncastle Soke | High Toynton | 68 |
| South Lindsey | Horncastle Soke | Horncastle | 1841 |
| South Lindsey | Horncastle Soke | Langriville | 29 |
| South Lindsey | Horncastle Soke | Low Toynton | 83 |
| South Lindsey | Horncastle Soke | Mareham le Fen | 428 |
| South Lindsey | Horncastle Soke | Mareham on the Hill | 90 |
| South Lindsey | Horncastle Soke | Moorby | 90 |
| South Lindsey | Horncastle Soke | Roughton with Haltham | 170 |
| South Lindsey | Horncastle Soke | Thimbleby | 186 |
| South Lindsey | Horncastle Soke | West Ashby | 349 |
| South Lindsey | Horncastle Soke | Wildmore | 119 |
| South Lindsey | Horncastle Soke | Wilksby | 56 |
| South Lindsey | Horncastle Soke | Wood Enderby | 107 |
| South Lindsey | Louth-Eske | Alvingham | 192 |
| South Lindsey | Louth-Eske | Authorpe | 199 |
| South Lindsey | Louth-Eske | Burwell | 141 |
| South Lindsey | Louth-Eske | Castle Carlton | 42 |
| South Lindsey | Louth-Eske | Conisholme | 157 |
| South Lindsey | Louth-Eske | Gayton le Wold | 52 |
| South Lindsey | Louth-Eske | Grainthorpe | 322 |
| South Lindsey | Louth-Eske | Great Carlton | 221 |
| South Lindsey | Louth-Eske | Grimoldby | 226 |
| South Lindsey | Louth-Eske | Haugham | 64 |
| South Lindsey | Louth-Eske | Keddington | 234 |
| South Lindsey | Louth-Eske | Kelstern | 158 |
| South Lindsey | Louth-Eske | Little Carlton | 108 |
| South Lindsey | Louth-Eske | Louth | 4067 |
| South Lindsey | Louth-Eske | Manby | 157 |
| South Lindsey | Louth-Eske | Muckton | 121 |
| South Lindsey | Louth-Eske | North Cockerington | 156 |
| South Lindsey | Louth-Eske | North Elkington with South Elkington | 294 |
| South Lindsey | Louth-Eske | North Reston | 53 |
| South Lindsey | Louth-Eske | North Somercoates | 512 |
| South Lindsey | Louth-Eske | Raithby with Hallington | 19 |
| South Lindsey | Louth-Eske | Ruckland with Farforth and Maidenwell | 82 |
| South Lindsey | Louth-Eske | Saltfleetby All Saints | 147 |
| South Lindsey | Louth-Eske | Saltfleetby St Clement | 109 |
| South Lindsey | Louth-Eske | Saltfleetby St Peter | 118 |
| South Lindsey | Louth-Eske | Skidbrooke with Saltfleetby Haven | 331 |
| South Lindsey | Louth-Eske | South Cockerington | 178 |
| South Lindsey | Louth-Eske | South Somercotes | 214 |
| South Lindsey | Louth-Eske | Stewton | 63 |
| South Lindsey | Louth-Eske | Tathwell | 235 |
| South Lindsey | Louth-Eske | Welton le Wold | 102 |
| South Lindsey | Louth-Eske | Withcall | 73 |
| South Lindsey | Louth-Eske | Yarburgh | 181 |
| South Lindsey | Wraggoe | Apley | 68 |
| South Lindsey | Wraggoe | Bardney | 674 |
| South Lindsey | Wraggoe | Benniworth | 226 |
| South Lindsey | Wraggoe | Biscathorpe | 37 |
| South Lindsey | Wraggoe | Burgh on Bain | 107 |
| South Lindsey | Wraggoe | East Barkwith | 192 |
| South Lindsey | Wraggoe | East Torrington | 92 |
| South Lindsey | Wraggoe | Goltho | 136 |
| South Lindsey | Wraggoe | Hainton | 172 |
| South Lindsey | Wraggoe | Hatton | 98 |
| South Lindsey | Wraggoe | Kirmond le Mire | 45 |
| South Lindsey | Wraggoe | Langton by Wragby | 153 |
| South Lindsey | Wraggoe | Legsby | 185 |
| South Lindsey | Wraggoe | Lissington | 126 |
| South Lindsey | Wraggoe | Ludford Magna with Ludford Parva | 318 |
| South Lindsey | Wraggoe | Panton | 154 |
| South Lindsey | Wraggoe | Rand | 104 |
| South Lindsey | Wraggoe | Sixhills | 292 |
| South Lindsey | Wraggoe | Snelland | 92 |
| South Lindsey | Wraggoe | Sotby | 77 |
| South Lindsey | Wraggoe | South Willingham | 227 |
| South Lindsey | Wraggoe | Stainfield | 179 |
| South Lindsey | Wraggoe | Stainton by Langworth | 228 |
| South Lindsey | Wraggoe | West Barkwith | 68 |
| South Lindsey | Wraggoe | West Torrington | 85 |
| South Lindsey | Wraggoe | Wickenby | 127 |
| South Lindsey | Wraggoe | Wragby | 391 |
| West Lindsey | Aslacoe | Blyborough | 122 |
| West Lindsey | Aslacoe | Caenby | 146 |
| West Lindsey | Aslacoe | Cammeringham | 110 |
| West Lindsey | Aslacoe | Coates | 42 |
| West Lindsey | Aslacoe | Cold Hanworth | 45 |
| West Lindsey | Aslacoe | Fillingham | 300 |
| West Lindsey | Aslacoe | Glentham | 282 |
| West Lindsey | Aslacoe | Glentworth | 236 |
| West Lindsey | Aslacoe | Hackthorn | 201 |
| West Lindsey | Aslacoe | Harpswell | 102 |
| West Lindsey | Aslacoe | Hemswell | 205 |
| West Lindsey | Aslacoe | Ingham | 173 |
| West Lindsey | Aslacoe | Normanby by Spital | 297 |
| West Lindsey | Aslacoe | Norton | 235 |
| West Lindsey | Aslacoe | Spridlington | 169 |
| West Lindsey | Aslacoe | Willoughton | 274 |
| West Lindsey | Corringham | Blyton cum Wharton | 394 |
| West Lindsey | Corringham | Corringham | 410 |
| West Lindsey | Corringham | Gainsborough [All Saints] | 5346 |
| West Lindsey | Corringham | Grayingham | 132 |
| West Lindsey | Corringham | Heapham | 133 |
| West Lindsey | Corringham | Kirton in Lindsey | 979 |
| West Lindsey | Corringham | Lea | 293 |
| West Lindsey | Corringham | Northorpe | 117 |
| West Lindsey | Corringham | Pilham | 89 |
| West Lindsey | Corringham | Scotter | 610 |
| West Lindsey | Corringham | Scotton | 379 |
| West Lindsey | Corringham | Springthorpe | 181 |
| West Lindsey | Lawress | Barlings | 54 |
| West Lindsey | Lawress | Brattleby | 191 |
| West Lindsey | Lawress | Broxholme | 160 |
| West Lindsey | Lawress | Burton by Lincoln | 254 |
| West Lindsey | Lawress | Buslingthorpe | 44 |
| West Lindsey | Lawress | Cherry Willingham | 77 |
| West Lindsey | Lawress | Dunholme | 139 |
| West Lindsey | Lawress | Faldingworth | 228 |
| West Lindsey | Lawress | Fiskerton | 288 |
| West Lindsey | Lawress | Freisthorpe | 44 |
| West Lindsey | Lawress | Greetwell | 88 |
| West Lindsey | Lawress | Nettleham | 337 |
| West Lindsey | Lawress | North Carlton | 95 |
| West Lindsey | Lawress | Reepham | 286 |
| West Lindsey | Lawress | Saxilby with Ingleby | 427 |
| West Lindsey | Lawress | Scampton | 165 |
| West Lindsey | Lawress | Scothern | 236 |
| West Lindsey | Lawress | Snarford | 45 |
| West Lindsey | Lawress | South Carlton | 155 |
| West Lindsey | Lawress | Sudbrooke | 89 |
| West Lindsey | Lawress | Welton | 320 |
| West Lindsey | Manley | Alkborough | 250 |
| West Lindsey | Manley | Appleby | 423 |
| West Lindsey | Manley | Bottesford | 578 |
| West Lindsey | Manley | Broughton | 540 |
| West Lindsey | Manley | Burton Stather | 505 |
| West Lindsey | Manley | Flixborough | 191 |
| West Lindsey | Manley | Frodingham | 436 |
| West Lindsey | Manley | Hibaldstow | 282 |
| West Lindsey | Manley | Messingham with East Butterwick | 734 |
| West Lindsey | Manley | Redbourne | 231 |
| West Lindsey | Manley | Roxby cum Risby | 339 |
| West Lindsey | Manley | Scawby | 520 |
| West Lindsey | Manley | Waddingham | 443 |
| West Lindsey | Manley | West Halton | 339 |
| West Lindsey | Manley | Whitton | 201 |
| West Lindsey | Manley | Winteringham | 489 |
| West Lindsey | Manley | Winterton | 661 |
| West Lindsey | Well | Gate Burton | 131 |
| West Lindsey | Well | Kettlethorpe with Laughterton and Fenton | 353 |
| West Lindsey | Well | Knaith | 64 |
| West Lindsey | Well | Marton | 278 |
| West Lindsey | Well | Newton on Trent | 217 |
| West Lindsey | Well | Stow in Lindsey | 552 |
| West Lindsey | Well | Torksey | 317 |
| West Lindsey | Well | Upton with Kexby | 339 |
| West Lindsey | Well | Willingham | 290 |
|  |  |  |  |
|  |  |  |  |
|  |  |  |  |
